# Computational perspective on structure, energetics, and bonding of FeC_2_O isomers with astrochemical significance

**DOI:** 10.3389/fchem.2026.1874752

**Published:** 2026-08-14

**Authors:** Abhirami R. J., Senthur Pandi Rajasabai

**Affiliations:** Department of Physics, School of Advanced Sciences, Vellore Institute of Technology, Vellore, Tamil Nadu, India

**Keywords:** bonding analysis, density functional theory, density of states, interstellar medium, Iron–bearing molecules, missing iron, spectroscopic parameters, astrochemistry

## Abstract

Iron in its elemental form has rarely been detected in the interstellar medium despite being the most abundant refractory element. The presence of iron oxides in the interstellar medium and the recent discovery of FeC in the circumstellar envelope of IRC+10,216 suggest iron-carbon-oxygen molecules as potential interstellar species. In light of this possibility, the potential energy surface of FeC_2_O has been investigated computationally using different levels of density functional theory calculations, yielding fourteen, seven, and twenty isomers across the quintet, triplet, and singlet electronic states, respectively. Within the examined DFT frameworks, the lowest energy isomer of FeC_2_O is in the quintet electronic state with a linear structure. Single-point (U)CCSD(T) calculations with T_1_ diagnostics reveal considerable multireference character in certain geometries. CASSCF optimizations are performed on quintet state geometries to account for the multireference character. Various computational tools, such as adaptive natural density partitioning (AdNDP) analysis, molecular orbital (MO) analysis, and Wiberg Bond Indices (WBI), are employed to elucidate the bonding characteristics of the global minimum geometry. The spectroscopic parameters in both infrared and microwave domains have been successfully computed. The total and the partial density of states are plotted to evaluate how different atoms contribute to the electronic structure. The structural, energetic, and spectroscopic parameters presented in this work have significant implications for future astronomical research.

## 1 Introduction

The interstellar medium (ISM) is of great importance to astrophysicists and astrochemists, as it encompasses a wide range of matter, from ions and atoms to molecules and dust grains. The composition includes interstellar clouds, star-forming regions, and planetary systems (Watson, 1977). Dense molecular clouds do not undergo ionization due to the absence of stellar radiation, leading to the predominance of molecular species. Conversely, diffuse interstellar clouds consist primarily of atoms because stellar radiation dissociates molecules. The grains in dense clouds consist of a silicate or graphite core enclosed in a mantle of various ices, mainly water ice (Herbst, 1995).

There have been numerous investigations into the composition of the ISM. Nearly 340 molecules have been identified in the ISM or circumstellar envelopes. Molecular hydrogen is the most abundant species, followed by carbon monoxide (Endres et al., 2016; Herbst, 2001). Iron, the sixth-most-abundant element, notably affects stellar nucleosynthesis and the life cycles of stars. Iron is obtained from Type Ia supernovae, core–collapse supernovae, and outflows emitted by Asymptotic Giant Branch (AGB) stars (Temple et al., 2020; Matteucci and Greggio, 1986). Iron lines are one of the significant components in the spectra of quasars (Temple et al., 2020; Matteucci, F. and Greggio, 1986; Yadav et al., 2023; Satyapal et al., 2021; Negus et al., 2021; Rose et al., 2015) and active galactic nuclei (AGNs) (Dong et al., 2008; Rodríguez-Ardila et al., 2004; Rubin et al., 1997). They are also found in the photoionized H II regions (Rodríguez, 2002). The atomic processes related to iron have always fascinated researchers, as they are used in astrophysical and laboratory plasmas (Schippers et al., 2017; Schippers et al., 2021; Beerwerth et al., 2019; Schippers and Müller, 2020; Olthof and Pottasch, 1975). Though Type Ia supernovae produce large amounts of Fe atoms, Fe grain formation has not yet been observed due to the ineffective homogeneous nucleation of iron grains in the interstellar environment (Kimura et al., 2017). Iron is strongly depleted from the gas phase into solids, with over 90% in dust, mostly in silicates (Dwek, 2016; Zhukovska et al., 2018). Iron missing from the ISM can precipitate onto pre-existing carbon or composite grains, potentially being sequestered as an impurity or incorporated into other particles, such as hydrogenated iron nanoparticles (Bilalbegović et al., 2017; Kimura et al., 2017). The missing iron can also be attributed to the formation of Fe–H nanoparticles by the interaction of iron atoms with hydrogen (Bilalbegović et al., 2017).

Most of the dust in the ISM is composed of five elements: C, O, Mg, Si, and Fe (Tielens and Allamandola, 1987). Only three chemical species containing iron have been identified in the ISM so far: FeCN (Zack et al., 2011), FeC (Koelemay and Ziurys, 2023), and FeO (Furuya et al., 2003; Merer et al., 1982; Walmsley et al., 2002), though it is the main refractory element prevalent there. Hence, the quest for further iron species in the ISM is imperative. The facts that oxygen and carbon are the third and fourth most prevalent components in the ISM, and iron is an abundant dust species only at the carbon–to–oxygen abundance ratio close to unity (Jones, 1990) indicate the possibility that iron can kinetically nucleate with carbon and oxygen, forming iron–carbon–oxygen systems in the ISM. Carbon is widely distributed in the ISM as atomic carbon (Sofia et al., 1997). It can exist in different forms, ranging from simple molecules such as carbon monoxide (CO) (Wilson et al., 1970; Solomon et al., 1971; Smith and Stecher, 1971) to complex organic compounds (Duley and Williams, 1981; Herbst and Van Dishoeck, 2009). The presence of CO, which forms the basis of interstellar ice, also increases the likelihood of detecting iron, carbon, and oxygen systems in the ISM. Moreover, ISM contains significant amounts of oxygen in solid–state components, such as dust and ice, and in gas–phase species, such as atoms and molecules, to support an oxygen–rich chemistry (Jensen, et al., 2008).

The presence of FeC radical was observed in the envelope of IRC+10216 (Koelemay and Ziurys, 2023). As FeC has the potential to function as a precursor species for FeCN, it may also facilitate the formation of Fe–C–O systems, which have yet to be explored. Also, iron oxide is likely to be a substantial part of interstellar grains. Investigations carried out by Jones (Jones, 1990) about iron and iron oxides within the ISM suggests that the expulsion of iron–enriched particulates into the interstellar environment, characterized by a relatively high concentration of atomic oxygen, will lead to their rapid conversion into iron (II) oxide and, subsequently, into magnetite. Another study to provide evidence suggests that iron oxides like maghemite (γ–Fe_2_O_3_) or magnetite (Fe_3_O_4_) cause the 21 μm infrared (IR) emission band (Cox, 1990). These possibilities also indicate the existence of Fe–C–O systems in ISM. Halfen *et al.* noted that FeCO^+^ is likely to be present in molecular clouds, where CO is highly concentrated, and that gas–phase iron is expected to be in the form of Fe^+^ (Halfen and Ziurys, 2007).

Although pure iron, its oxides and sulfides, and hydrogenated iron nanoparticles are discussed as components of interstellar dust grains (Bilalbegović et al., 2017; Merer et al., 1982; Jones, 1990; Cox, 1990; Köhler et al., 2014), iron–carbon–oxygen systems have not been studied extensively in the astrophysical literature. Hence, the present work aims to explore the potential energy surface (PES) of iron–carbon–oxygen systems in the ISM, focusing on systems containing one iron, two carbon, and one oxygen atom, using the density functional theory (DFT). A computational study of the equilibrium geometries, binding energies, and electronic structures of neutral FeC_n_ clusters (n < 3) concluded that DFT provides a reliable method for explaining experimental outcomes and can be used to accurately examine larger FeC_n_ systems, especially when other quantum chemical methods are computationally challenging (Nash et al., 1996). Also, DFT simulations were used to investigate the structural, electronic, vibrational, and rotational properties of small cyclic molecules containing iron, carbon, and hydrogen (Chandra, 2013). The dicationic and neutral species of FeC_4_H_2_ have also been examined, with implications for astrophysical contexts; extensive investigations and analyses have been conducted on iron, carbon, and hydrogen systems that closely resemble the earlier findings (Shajan et al., 2024; Shajan and Thirumoorthy, 2024).

By comparing radio astronomical data with laboratory rotational spectra, it is possible to accurately identify most molecules in the ISM. However, identifying, characterizing, and synthesizing such transitory species in the laboratory is complicated. Consequently, theoretical studies of the chemical composition and electronic properties of these critical molecules in astrophysics could aid in detecting them in the ISM and the laboratory for radioastronomers and molecular spectroscopists. In this context, the current study provides structural and spectroscopic data of FeC_2_O isomers in the neutral state using DFT. The obtained data could support subsequent molecular spectroscopic observations and radio astronomical searches (Roy et al., 2021; Satpati et al., 2023a; Satpati et al., 2023b; Thirumoorthy et al., 2018). Investigating the structure, energetics, bonding, and spectroscopic properties of these molecules will be advantageous for future astronomical observations. The formation of FeC_2_O in ISM is expected to be difficult from an astrochemical perspective. Although a detailed kinetic analysis is beyond the scope of the present study, a few feasible gas-phase formation pathways are qualitatively discussed based on known ISM conditions. Identifying FeC_2_O in the ISM will deepen our understanding of metal–carbon chemistry, illuminate catalytic processes in molecular clouds, and help account for the missing iron in the ISM.

## 2 Computational methodology

The isomers of FeC_2_O are explored in singlet, triplet, and quintet electronic states in the neutral charge state. Chemical intuition is used to generate preliminary geometries of the FeC_2_O system. Density functional theory has been used with and without an effective core potential for optimizations. The methods used are TPSSh (Becke, 1988; Cirera et al., 2018) and ωB97XD (Chai and Head-Gordon, 2008; Grimme et al., 2010), employing the basis sets 6–311++G (2d,2p) (Chan, 2023; Krishnan et al., 1980; Clark et al., 1983; Pham and Nguyen, 2015; Tam et al., 2015; Louis et al., 2023) and SDD (Kreutzer et al., 2016; Bergner et al., 1993; Szilagyi and Winslow, 2006). The quintet and triplet states are treated using the unrestricted formalism. TPSSh is a hybrid functional derived from the TPSS functional (Tao–Perdew–Staroverov–Scuseria) (Tao et al., 2003) and contains a 10% exact-exchange contribution (Marzouk et al., 2011), whereas the ωB97XD functional includes long–range and empirical dispersion corrections (Grimme et al., 2010; Panda et al., 2022). The 6–311++G (2d,2p) and the combination of SDD and 6–311++G (2d,2p) basis sets have been used with both methods for optimization and frequency calculations. The iron atom has been treated with SDD (Zayed et al., 2019; Moustafa et al., 2020), while the 6–311++G (2d,2p) basis set has been utilized for C and O atoms (Clark et al., 1983). The Stuttgart/Dresden (SDD) method accounts for the relativistic effects in the Fe atom (Fuentealba et al., 1982; Szilagyi and Winslow, 2006).

Geometry optimizations and frequency calculations have been performed using the Gaussian 16 program (Frisch et al., 2016). Harmonic vibrational frequencies are calculated for each optimized geometry in order to determine whether the stationary point on the PES is a minimum, transition state, or higher–order saddle point. Wavefunction stability analyses are performed for the optimized triplet and quintet minima, confirming convergence to stable electronic solutions.

To assess the sensitivity of the DFT energy ordering to a correlated wavefunction approach, single-point (U)CCSD(T) (Purvis and Bartlett, 1982; Raghavachari et al., 2013) calculations were carried out on ωB97XD/6–311++G (2d,2p) optimized geometries. T_1_ diagnostic values are also calculated for all quintet, triplet, and singlet isomers at the (U)CCSD(T)/6–311++G (2d,2p)//UωB97XD/6–311++G-(2d,2p) level of theory in order to evaluate the multireference character (Lee and Taylor, 1989), following the approach adopted in recent computational studies of metal systems from an astrochemical perspective (Roy et al., 2024). Moreover, the quintet isomers are further optimized at the UCCSD/6–311++G (2d,2p) level to verify the robustness of the quintet energetic ordering beyond single-point comparisons. To account for the multireference character of the quintet isomers, complete active space self-consistent field (CASSCF) (Bjorn and Ross, 1980; Roos, 1980) optimizations have been performed with an active space of (10,10) and def2-tzvp basis set using ORCA software (Neese et al., 2020).

The spectroscopic study, including IR spectra and calculations of rotational and centrifugal distortion constants, has been performed using Gaussian 16 at the UωB97XD/6–311++G (2d,2p) level of theory. The rotational and centrifugal distortion constants have been derived from the harmonic force field through vibration–rotation analysis.

Further, the chemical bonding characteristics of the FeC_2_O global minimum have been studied at the UωB97XD/6–311++G (2d,2p) level of theory, which was chosen for the detailed bonding analysis due to its balanced description of Fe-C and Fe-O interactions and its appropriateness for charge-transfer effects (Jacquemin et al., 2011; Berryman et al., 2014; Shajan et al., 2024; Shajan and Thirumoorthy, 2024). The chemical bonding characteristics of the global minimum geometry of FeC_2_O in the quintet electronic state are comprehensively characterized by using quantum chemical tools such as adaptive natural density partitioning (AdNDP) analysis (Osorio, 2023; Zubarev and Boldyrev, 2008), molecular orbital (MO) analysis (Prabhaharan et al., 2014), and Wiberg bond indices (WBIs) (Wiberg, 1968). Additionally, the natural atomic charges (NAC) (Glendening et al., 1998; Reed et al., 1985) derived from Natural Bond Orbital (NBO) analysis (Reed et al., 1985), Electron Localization Function (ELF) (Becke and Edgecombe, 1990; Burdett and McCormick, 1998; Savin et al., 1997), Atoms in Molecules (AIM) (Matta et al., 2007; Bader, 1991), and the Laplacian of the electron density (Mierzwa et al., 2015) are provided in the Supplementary Material. Estimation of WBIs, NACs, and MO analysis has been performed using Gaussian 16, while analysis of the Laplacian of the electron density, bond critical points, and the density of states (DOS) plot has been carried out using the Multiwfn program (Lu and Chen, 2012).

## 3 Results and discussion

**FIGURE 1:**
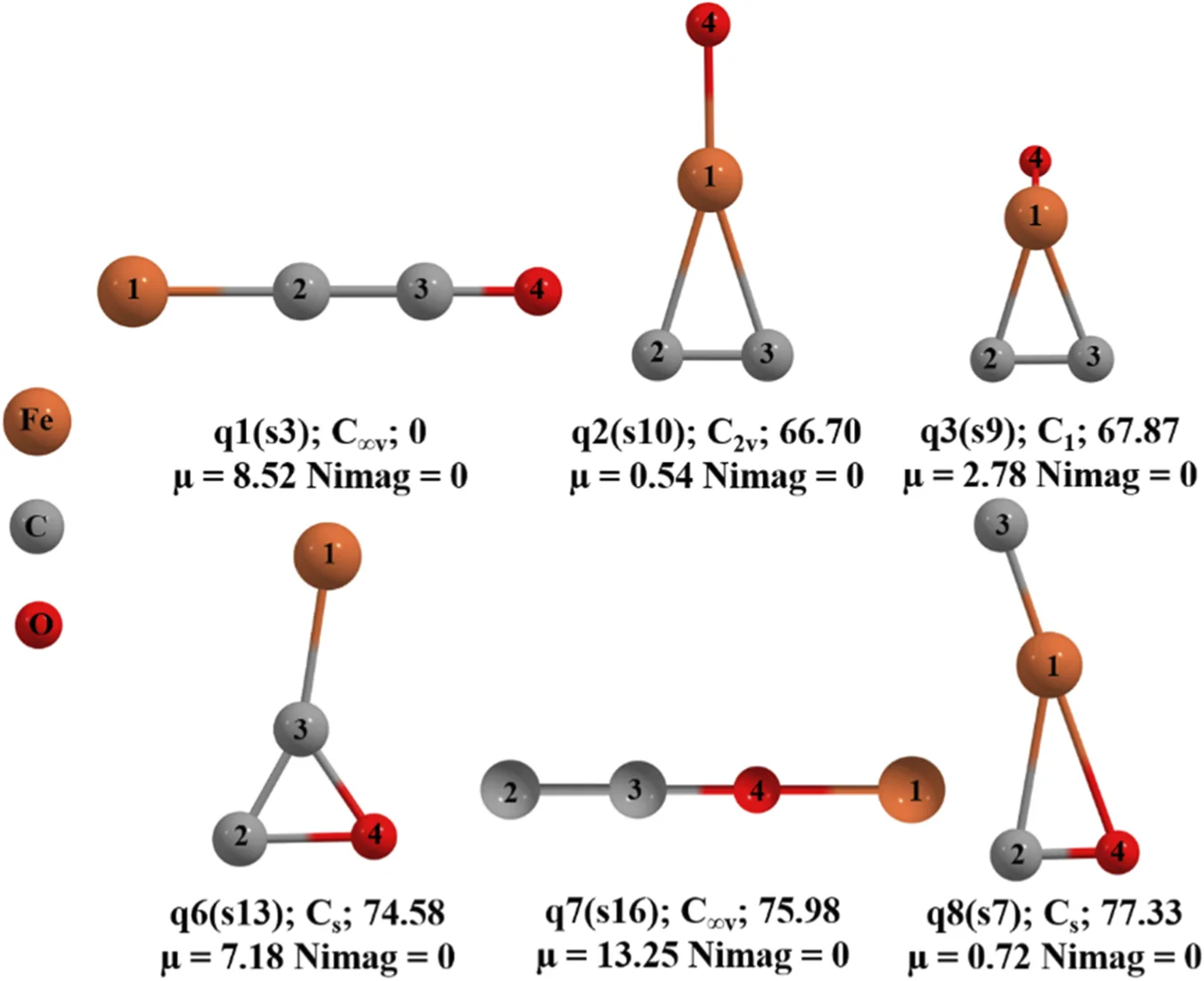
Isomers of FeC_2_O representing minima (without imaginary frequencies) in their corresponding quintet electronic states, with their point group, ZPVE corrected relative energies (in kcal mol^–1^), dipole moment (in Debye), and number of imaginary frequencies (Nimag) obtained at the UωB97XD/6–311++G (2d,2p) theoretical level. The corresponding singlet states are included in parentheses.

The PES of FeC_2_O in the quintet electronic state has been scrutinized using different theoretical frameworks, as noted earlier. The results obtained by using the different theoretical frameworks: UTPSSh/6–311++G (2d,2p), UTPSSh/SDD&6–311++G (2d,2p), UωB97XD/6–311++G (2d,2p) and UωB97XD/SDD&6–311++G (2d,2p) for the quintet electronic state are compared and analyzed. The geometric optimization culminated in the identification of fourteen distinct isomers of FeC_2_O in its quintet state. Although a preliminary extensive investigation of the PES revealed multiple stationary points, vibrational analysis suggests that only a few correspond to true minima. At the UωB97XD/6–311++G (2d,2p) level of theory, six isomers (q1, q2, q3, q6, q7, and q8) correspond to minima, and four (q4, q5, q9, and q10) pertain to transition states and higher-order saddle points. The discussion is hence confined to these experimentally significant structures shown in Figure 1. The isomers with non-zero imaginary frequencies are shown in Supplementary Figure S1 in the Supplementary Material. Beneath the geometry, the point group symmetries, total energy, permanent dipole moments, and the number of imaginary frequencies (Nimag) for each stationary point are displayed. The isomers are designated by their spin multiplicity (q = quintet, t = triplet, s = singlet) and are sequentially numbered according to their respective energies within each spin state. Comparisons among various spin states are primarily based on structural similarity rather than numerical designations. The optimized geometries of FeC_2_O isomers in the quintet state, which are unattainable with the UωB97XD/6–311++G (2d,2p) level, but achievable with the UTPSSh/6–311++g (2d,2p), UTPSSh/SDD&6–311++G (2d,2p), or UωB97XD/SDD&6–311++G (2d,2p) theoretical levels are presented in Supplementary Figures S2–S4 in the Supplementary Material. The geometry optimization of FeC_2_O in its triplet electronic state performed at the UωB97XD/6–311++G (2d,2p) theoretical level yielded seven isomers, which are shown in Supplementary Figure S5 of the Supplementary Material. Supplementary Figure S6 in the Supplementary Material shows the sixteen isomers of FeC_2_O in the singlet electronic state, optimized at the ωB97XD/6–311++G (2d,2p) level, and Supplementary Figures S7–S9 show FeC_2_O isomers in the singlet state, which are unattainable with the ωB97XD/6–311++G (2d,2p) level, but attainable with other levels of theory. The point group, total energy (in a. u), zero point correction (in a. u), zero point vibrational energy (ZPVE) corrected total energy (E + ZPVE; in a. u), relative energy with ZPVE corrections (ΔE + ZPVE; in a. u), relative energies with ZPVE corrections (ΔE + ZPVE) in kcal mol^–1^, absolute dipole moment (in Debye) and the number of imaginary frequencies (Nimag) for FeC_2_O in its quintet state optimized at the different levels of theories can be found in Supplementary Tables S1–S4 in the Supplementary Material. Supplementary Table S5 presents the details for FeC_2_O isomers in the triplet electronic state at the UωB97XD/6–311++G (2d,2p) level of theory, while Supplementary Tables S6–S9 provide the specifics for FeC_2_O singlets optimized at the different levels of theory. Also, Supplementary Table S10 compares the ZPVE corrected relative energies of FeC_2_O isomers (in kcal mol^–1^) in their corresponding singlet electronic states obtained at different levels.

### 3.1 Structure and energetics

The number of FeC_2_O isomers identified in the quintet electronic state depends on the level of theory employed. The maximum number of isomers of FeC_2_O in the quintet electronic state, totalling thirteen, is obtained at UTPSSh/SDD and 6–311++G (2d,2p) theoretical level, followed by twelve isomers found at the UTPSSh/6–311++G (2d,2p) theoretical level. Using the UωB97XD method, ten isomers are obtained without SDD, and nine with SDD. Isomer q1 shown in Figure 1 is the global minimum for FeC_2_O in the quintet electronic state found at the UωB97XD/6–311++G (2d,2p) level of theory. The same linear structure is obtained as the minimum-energy structure at the UωB97XD/SDD and 6–311++G (2d,2p) level of theory also. In contrast, the UTPSSh method yields isomer q11 as the lowest-energy structure, with or without SDD. The energy difference between q11 and q1 in these methods is very minimal. All levels of theory employed in this study consistently provide isomers q1, q2, q4, q5, q6, q7, and q10, implying that these structures represent robust portions of the PES. The UTPSSh/SDD and 6–311++G (2d,2p) level introduces an additional highest-energy isomer q14, which is not obtained at the other levels of theory. It resembles q5, with slightly elongated peripheral bonds and an absence of a C-C bond. At the UωB97XD/6–311++G (2d,2p) level of theory, both the lowest- and highest-energy quintet isomers are linear structures. The lowest energy is achieved when Fe is terminally bonded to C2, whereas the maximum energy is achieved when Fe comes between C2 and C3. Also, linear structures with terminal Fe atoms are minima, while those with Fe in the middle have two imaginary frequencies. Isomer q11 is obtained at all the theoretical levels except the UωB97XD/6–311++G (2d,2p) level of theory. Isomers q12 and q13 are obtained only with the UTPSSh method. Table 1 compares the ZPVE–corrected relative energies of FeC_2_O isomers in the quintet electronic state, calculated using different computational methods. The optimized geometrical parameters, bond lengths in angstroms (Å), and bond angles in degrees for FeC_2_O isomers in their quintet and singlet electronic states, computed at different levels of theory, are respectively tabulated in Supplementary Tables S11, S12 in the Supplementary Material.

**TABLE 1:** ZPVE-corrected relative energies of FeC_2_O isomers (in kcal mol^–1^) in their corresponding quintet electronic states obtained at different levels. The isomers are arranged in the order of energy computed at the UωB97XD/6–311++G (2d,2p) level of theory.

Isomers	ZPVE corrected relative energy in kcal mol^–1^			
	UωB97XD/6–311++G (2d,2p)	UTPSSh/6–311++G (2d,2p)	UTPSSh/SDD and 6–311++G (2d,2p)	UωB97XD/SDD and 6–311++G (2d,2p)
q1	0	0.34	1.84	0
q2	66.7	60.38	81.65	79.17
q3	67.87	55.31	63.3	NA
q4	69.66	68.15	75.36	71.53
q5	74.29	74.04	82.99	92.13
q6	74.58	75.51	70.43	73.52
q7	75.98	65.04	56.52	75.17
q8	77.33	NA	NA	81.12
q9	78.71	74.99	76.56	NA
q10	85.48	55.62	61.1	69.91
q11	NA	0	0	4.93
q12	NA	48.88	54.69	NA
q13	NA	104.26	108.46	NA
q14	NA	NA	113.66	NA

* NA (not applicable) indicates that the geometry did not converge at that level.

The minimum energy geometry of FeC_2_O in the quintet and triplet electronic states is a similar linear structure, whereas it is different in the singlet electronic state. Wavefunction stability studies are conducted for the optimized quintet global minimum. Following orbital optimization, the final wavefunction was validated as stable against internal perturbations. The stability study verifies that the wavefunction represents a true minimum.

The ⟨S^2^⟩ value of q1 (6.001) approximates the ideal quintet value (6.00), signifying negligible spin contamination. A similar study carried out with the triplet minimum yielded an ⟨S^2^⟩ value post-spin annihilation of 2.27, indicating moderate spin contamination. Isomers t2, t3, and t6 show only minor deviations from the ideal triplet value (2.00). Nevertheless, a few triplet structures, such as t4, t5, and t7, exhibit noticeable spin contamination. Such contamination results from mixing higher-spin electronic configurations into the unrestricted wavefunction and may affect the quantitative relative energies of closely spaced triplet isomers. This is consistent with the UCCSD(T) results, where the low-spin-contaminated t2 structure is the triplet minimum rather than the t1 structure predicted by DFT. These observations, along with the T_1_ diagnostic and CASSCF investigations, imply that electron correlation and multiconfigurational effects are significant for the energy ordering of the triplet state. However, the very small spin contamination in q1, together with the correlated wavefunction calculations, suggests that the assignment of the quintet global minimum is unaffected.

**FIGURE 2:**
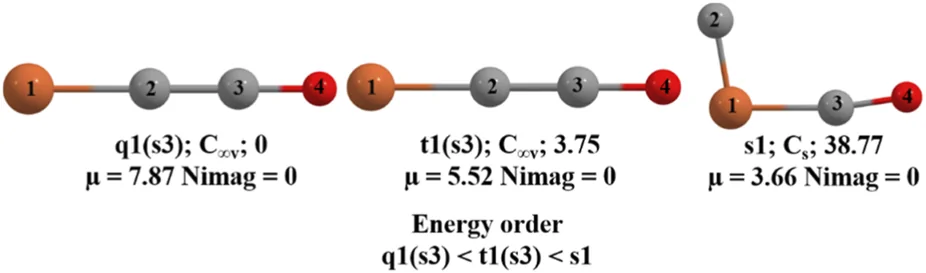
Energy order of the minimum energy geometry in the singlet, triplet, and quintet electronic states, including their point group, ZPVE corrected relative energies (in kcal mol^–1^) with respect to q1, dipole moment (in Debye), and number of imaginary frequencies (Nimag), obtained at the UωB97XD/6–311++G (2d,2p) level of theory for FeC_2_O. The corresponding positions of singlet states are included in parentheses.

A comparison of the minimum-energy geometries across the three electronic states reveals that the global minimum for FeC_2_O, q1, occurs in the quintet state and adopts a linear configuration akin to t1 and s3. The triplet counterpart of q1 possesses an energy elevation of merely 3.74 kcal/mol. The stability order of the minimum energy geometry in the singlet, triplet, and quintet electronic states, along with their point group, ZPVE corrected relative energies with respect to q1 (in kcal mol^–1^), the dipole moment (in Debye), and the number of imaginary frequencies (Nimag) obtained at the UωB97XD/6–311++G (2d,2p) level of theory for FeC_2_O are depicted in Figure 2.

To facilitate direct comparison of spin-state energetics among the identified isomers, the relative energies of all observed minima are compiled in Table 2. Energies are referenced to the global minimum (q1) across all spin configurations. For open-shell states, the ⟨S^2^⟩ values post-annihilation were validated. The calculated relative energy demonstrates a distinct dependence on spin states. Most of the isomers preferentially stabilize in the quintet state, with q1, q2, q3, q6, q8, and q5 exhibiting quintet ground states. In contrast, the isomers that did not converge in the quintet state (t2, t3, t6) prefer the triplet multiplicity. An exception is isomer q7. Isomers s2, s5, and s6 converged exclusively in the singlet state. The ⟨S^2^⟩ values for triplet (≈2.04–2.64) and quintet (≈6.00–6.02) states indicate generally acceptable spin purity, with minor spin contamination observed in selected cases. Overall, the results demonstrate that the preferred multiplicity strongly depends on the structure.

**TABLE 2:** ZPVE corrected relative energies of FeC_2_O isomers (in kcal mol^–1^) of the optimized structures of FeC_2_O across singlet, triplet, and quintet electronic states computed at the UωB97XD/6 311++G (2d,2p) level of theory.

Isomer	Multiplicity	ZPVE corrected relative energy (kcal/mol)	⟨S^2^⟩	Lowest energy multiplicity
q1	singlet^†^	60.58	-	Quintet
	Triplet	3.74	2.27	
	Quintet	0	6.00	
q2	singlet^†^	105.47	-	Quintet
	Quintet	66.70	6.02	
q3	Singlet	102.04	​	Quintet
	Quintet	67.87	6.00	
q6	Singlet	115.70	-	Quintet
	Quintet	74.58	6.00	
q7	singlet^‡^	133.35	-	Triplet
	triplet^‡^	52.46	2.64	
	Quintet	75.98	6.00	
q8	Singlet	78.83	-	Quintet
	Quintet	77.33	6.00	
t2	Singlet	38.77	-	Triplet
	Triplet	36.00	2.07	
t3	Singlet	62.32	-	Triplet
	Triplet	38.69	2.04	
t6	Singlet	112.15	-	Triplet
	Triplet	78.79	2.04	
s2	Singlet	57.53	-	Singlet
s5	Singlet	65.63	-	Singlet
s6	Singlet	77.36	-	Singlet
q5	Singlet	144.17	-	Quintet
	triplet^†^	75.43	2.37	
	quintet^†^	74.29	6.00	

Entries marked “—” indicate that a corresponding minimum was not located for that multiplicity. The superscript.

^†^ indicates transition states, and.

^‡^ indicates a higher-order saddle point.

**FIGURE 3:**
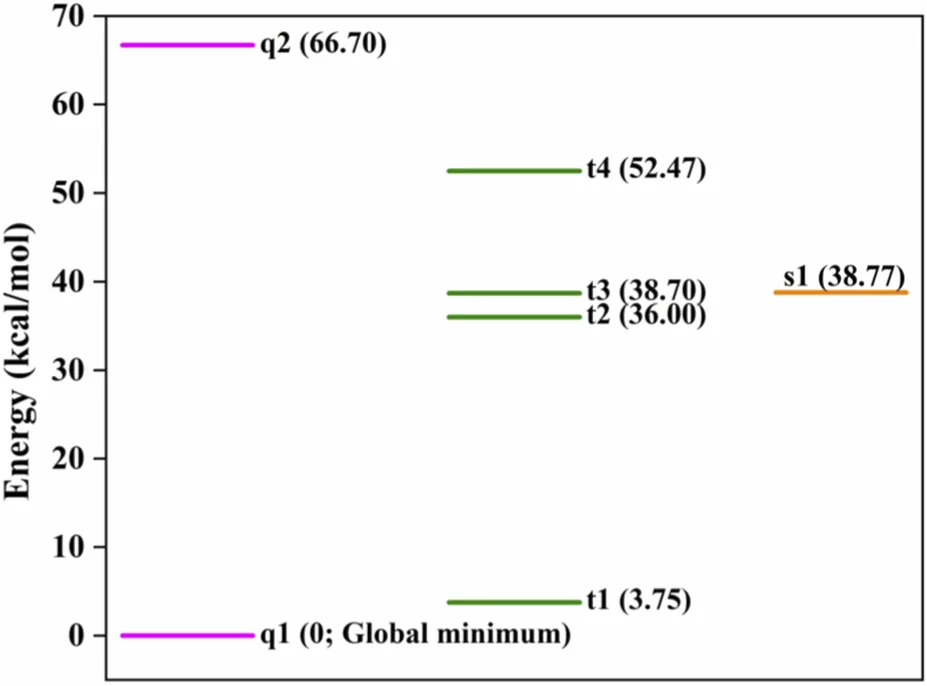
Relative energies of the low-lying FeC_2_O isomers in singlet (orange), triplet (green), and quintet (pink) spin states referenced to the global minimum (q1). Relative energies are given in parentheses in kcal mol^-1^; obtained at the (U)ωB97XD/6–311++G (2d,2p) level of theory.

To elucidate the energetic hierarchy of FeC_2_O isomers across various spin states, the relative energies of all singlet, triplet, and quintet configurations are benchmarked against the global minimum quintet isomer (q1). The resultant energy landscape of the isomers with energy below 70 kcal/mol is depicted in Figure 3. The triplet minimum is situated at merely 3 kcal mol^-1^ above q1, suggesting that other spin states may be energetically attainable. Conversely, the singlet structures exist at considerably elevated energy, indicating that they are inaccessible in frigid interstellar environments, although they may still be relevant for understanding the broader PES.

### 3.2 Correlated wavefunction assessment of the FeC_2_O PES

To analyze the sensitivity of DFT to a correlated wavefunction approach, single-point (U)CCSD(T)/6–311++G (2d,2p) calculations were implemented on the singlet, triplet, and quintet structures optimized at the UωB97XD/6–311++G (2d,2p) level of theory. Despite several energetic reorderings occurring at the single point (U)CCSD(T) level of calculations, the preference for the linear quintet structure q1 as the global minimum of FeC_2_O remains consistent, indicating that the ground-state assignment is unaffected by the treatment of electron correlation. However, the relative ordering of certain high-energy isomers is modified at the UCCSD(T) level. especially for low-lying open-shell states with significant multiconfigurational character, as further indicated by the T_1_ diagnostic values.

At the UTPSSh level, the linear quintet structure q1 exhibits a second-order saddle point, whereas q11 represents the minimum energy geometry. The relative energy difference between these structures at the UTPSSh/6–311++G (2d,2p) level of theory is only 0.34 kcal mol^-1^. Due to this disparity, additional single-point UCCSD(T)/6–311++G (2d,2p) calculations were performed on the UTPSSh-optimized geometries of q1 and q11. The CCSD(T) results indicate that q1 is lower in energy than q11 by 6.71 kcal mol^-1^, indicating the qualitative energetic preference for q1 over q11 at the correlated wavefunction level. These results further substantiate q1 as the global minimum geometry, despite the UTPSSh prediction of q11 as the minimum.

The most significant change occurs in the triplet state, where the bent structure t2 (like s1) becomes the triplet minimum, rather than the linear structure t1 predicted at the UωB97XD/6–311++G (2d,2p) level of theory. Isomer s1, with a bent structure, attains lower energy than its triplet structural counterpart t2. Also, certain high-energy quintet isomers, particularly q10, q4, and q7, exhibit significant stabilization, and further energetic shifts are observed for certain singlet isomers. These variations suggest that the FeC_2_O PES is sensitive to the treatment of electron correlation. Nevertheless, it has been observed that the lowest singlet (s1) and triplet (t2) structures, respectively, remain 16.22 and 18.02 kcal mol^-1^ above q1, indicating that the quintet ground state is well separated from the low-spin states. Consequently, while the energetic ordering of the low-lying excited states is method-dependent, q1 is consistently preserved as the global minimum structure.

Supplementary Figure S10 in the Supplementary Material compares the relative energy of the minimum energy geometry in the singlet, triplet, and quintet electronic states acquired from single-point energy calculations. The comparison of relative energies (ΔE + ZPVE) of isomers optimized at the (U)ωB97XD/6–311++G (2d,2p) level of theory, together with single-point energy calculations at the (U)CCSD(T) level and 6–311++G (2d,2p) basis sets, is presented in Supplementary Tables S13–S15.

The T_1_ diagnostic values were computed for the singlet, triplet, and quintet FeC_2_O isomers to examine the multireference nature and are reported in Supplementary Tables S13–S15 of the Supplementary Material. The calculated T_1_ values imply that the multireference character is significant across a large region of the FeC_2_O PES. In the quintet state, certain isomers, including q3, q4, q8, q9, and q10, have large T_1_ values, consistent with their pronounced energy shifts between DFT and UCCSD(T). The q7 isomer is an extreme case, with an exceptionally large T_1_ value, which implies that its electronic structure is poorly described by a single-reference wavefunction and that its UCCSD(T) relative energy should be interpreted with considerable caution. The triplet state also exhibits large multireference character, including the low-lying t1 and t2 structures, for which the ordering is reversed at the UCCSD(T) level. The singlet state also exhibits high T_1_ values, even for the lowest-energy singlet structures, providing further evidence that the low-lying FeC_2_O PES is not purely single-reference in nature. Therefore, the UCCSD(T) single-point energies are employed here primarily as qualitative correlated-wavefunction comparisons to evaluate energetic trends and spin-state ordering, rather than as definite quantitative reference values for the entire FeC_2_O PES.

To analyze the robustness of the quintet energetic ordering beyond single-point comparisons, the quintet isomers are further optimized at the UCCSD/6–311++G (2d,2p) level. Among the structures that converged successfully at this level (shown in Supplementary Figure S11 in the Supplementary Materials), q1 remained the lowest-energy quintet isomer, while q2, q4, and q5 remained higher in energy. Similarly, the higher energy structures q13 and q14 were identified well above q1. The UCCSD optimizations failed to converge for certain quintet isomers, consistent with the high electronic complexity and multireference sensitivity indicated by the T_1_ diagnostics and the UCCSD(T) energetic reordering. Although these CCSD optimizations do not provide a complete description of the quintet state, they further substantiate the stability of q1 as the preferred lowest-energy quintet FeC_2_O structure within the explored theoretical framework.

To further study the multireference character of the quintet isomers, as indicated by the T_1_ diagnostic values, CASSCF geometry optimizations have been performed. The CASSCF calculations also identify q1 as the lowest energy quintet structure. The relative energies of certain higher-energy quintet isomers are different from the DFT results, especially for q7. These differences are consistent with the considerable multiconfigurational character suggested by the T_1_ diagnostic values. Moreover, the energy difference between q1 and q11 at the CASSCF/def2-tzvp level of theory is negligible (0.000048 kcal/mol). Overall, the CASSCF calculations support the conclusion that multireference character alter the relative energies of several higher-energy isomers, but the identification of q1 as the preferred low-energy quintet structure remains consistent. Supplementary Tables S16, S17 in the Supplementary Material compare the relative energies of FeC_2_O quintet isomers obtained at the UωB97XD/6–311++g (2d,2p) with those obtained at the UCCSD/6–311++G (2d,2p) and the CASSCF/def2-tzvp levels of theory, respectively.

### 3.3 Dissociation pathways and intrinsic stability of q1

The local minima can be qualitatively considered as resulting from interactions between Fe and various C-O fragments (as Fe + C_2_O or FeC + CO). Open-chain linear structures correspond to end-on coordination of Fe with the C_2_O fragment, while cyclic or bending geometries result from side-on or perpendicular interactions that involve simultaneous Fe–C and Fe–O contacts. To determine the intrinsic stability of FeC_2_O, two fragmentation channels, Fe + C_2_O and FeC + CO, are studied on the quintet PES, associated with q1. At the UωB97XD/6–311++G (2d,2p) level of theory, the calculated bond dissociation energies are 216.3 and 338.9 kcal mol^-1^, respectively, indicating that FeC_2_O is strongly bound with respect to these dissociation pathways. Additionally, relaxed potential-energy scans are done along the Fe–C and C–C coordinates. They show a steady increase in energy with bond elongation, and no stationary points are observed. While detailed kinetic modeling and reaction-rate computations are beyond the scope of the present study, the large dissociation energies suggest that FeC_2_O is strongly bound relative to the investigated fragmentation channels, indicating its intrinsic stability that requires consideration in future astrochemical studies.

### 3.4 Bonding analysis

**FIGURE 4:**
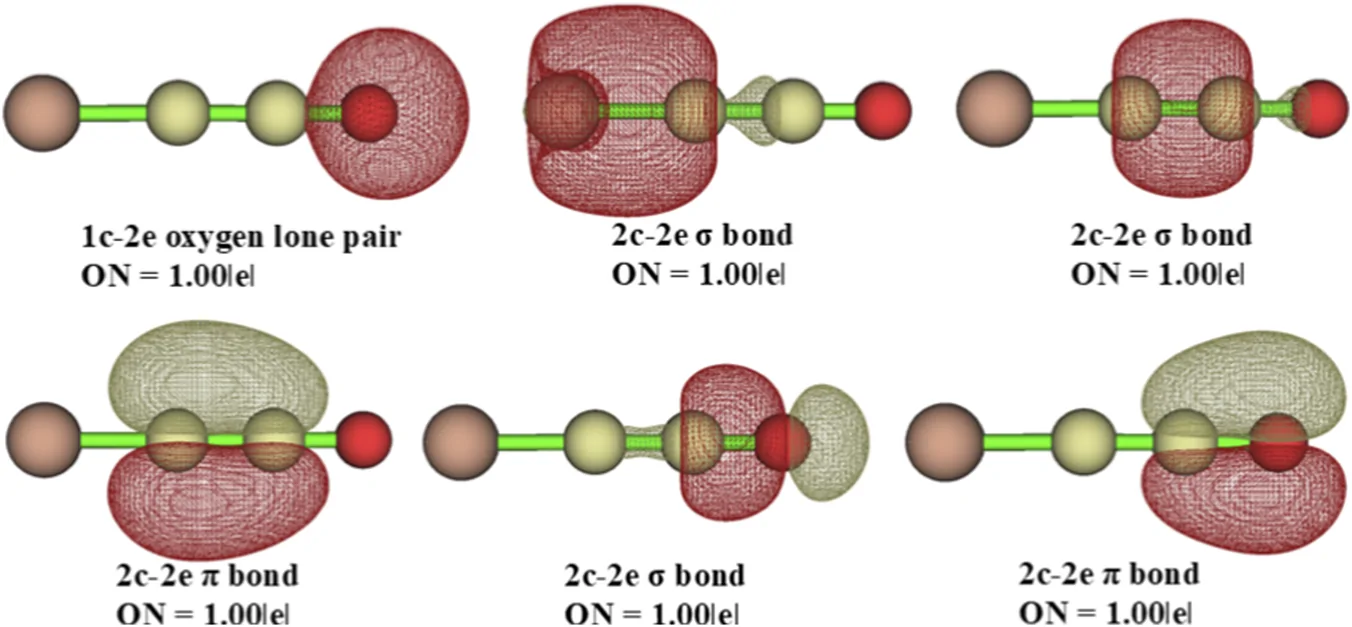
The AdNDP bonding patterns of q1 with occupation numbers (ONs) obtained at the UωB97XD/6–311++G (2d,2p) level.

To understand the nature of bonding within the isomers, AdNDP analysis has been performed on the alpha orbitals. This technique provides exhaustive information on both localized and delocalized bonding in molecules through wavefunction interpretation and thereby serves as a powerful enhancement of NBO for electron density. In AdNDP analysis, the occupancy number (ON) denotes the total number of electrons associated with a specific bonding element. The highest possible value for ON that describes the electronic structure in terms of *n-center* two–electron (*n*c−2e) bonds is two (Zubarev and Boldyrev, 2008). AdNDP analysis is particularly well-suited to systems with a high degree of electron delocalization, providing essential insights into lone pairs, aromaticity, delocalized bonding elements, and chemical bonding patterns. It is helpful for studying systems that are hardly characterized by conventional methods due to their complex bonding patterns. Figure 4 shows the α-spin AdNDP bonding patterns of q1, with ONs. It supports WBI and NAC analysis interpretations. The 2c–2e σ bonds, distinctly observed for Fe–C2, C2–C3, and C3–O, establish the essential structural linkage. The well–defined π bonds in both the C2–C3 and C3–O connections imply that each π interaction is predominantly concentrated between its corresponding atom pair in the high–spin state. This localization indicates minimal involvement of Fe d orbitals in π bonding, consistent with diminished backbonding in a quintet structure. The AdNDP orbitals collectively affirm that the characteristics of the C=C and C=O double bonds are preserved. At the same time, Fe predominantly provides σ-bonding support to the carbon core, consistent with the WBI and charge distributions. The β-spin AdNDP analyses are also conducted to verify the adequacy of the bonding description. Similar to α-spin analysis, β-spin analysis reproduces the fundamental bonding framework, encompassing localized Fe–C, C–C, and C–O bonding interactions, as well as oxygen lone-pair orbitals. Consequently, the qualitative bonding representation is unaffected by the chosen spin state. The β-spin AdNDP orbitals of q1 are shown in Supplementary Figure S12 in the Supplementary Material.

**FIGURE 5:**
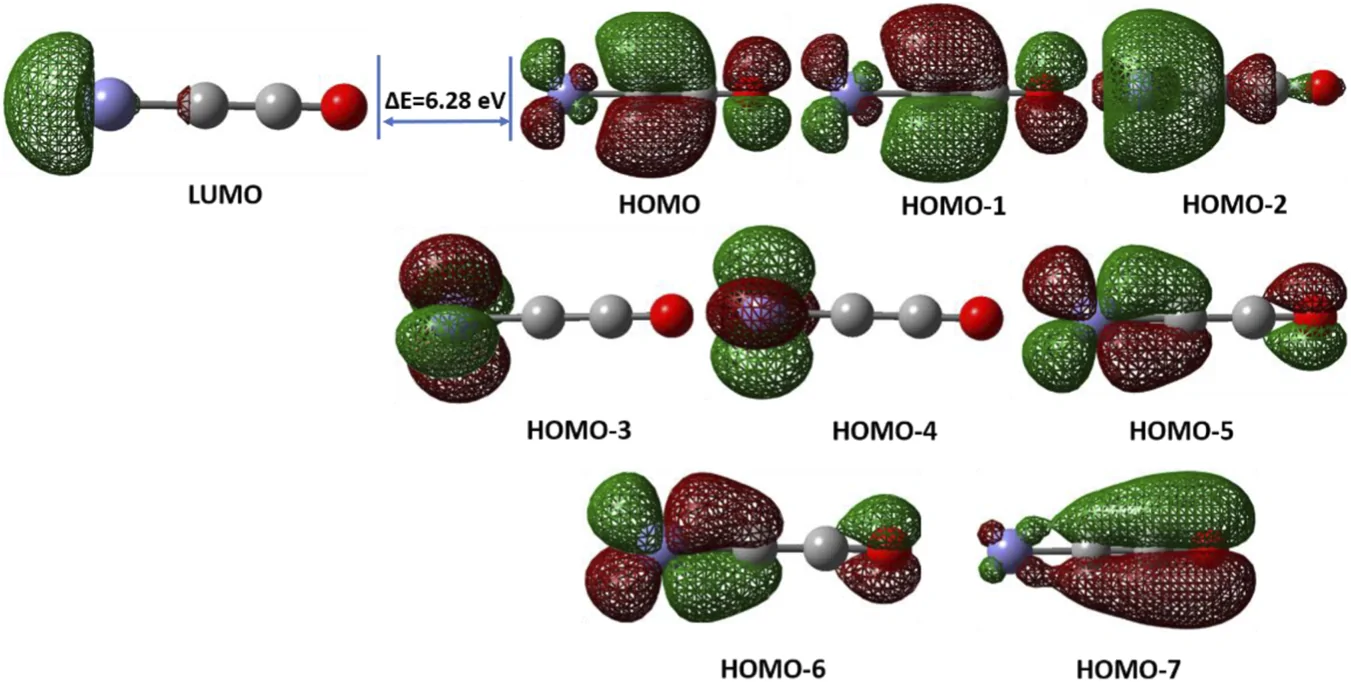
The molecular orbitals of q1 obtained at the UωB97XD/6–311++G (2d,2p) level (isovalue = 0.03).

MO analysis is conducted to substantiate the bonding interpretation derived from AdNDP. Figure 5 displays the lowest unoccupied molecular orbital (LUMO) and highest occupied molecular orbital (HOMO) alpha orbitals of q1. Here, HOMO, HOMO–1, HOMO–5, HOMO–6, and HOMO–7 are π delocalized orbitals, while HOMO–2 is a σ delocalized orbital. The d orbitals of Fe predominantly exhibit nonbonding characteristics, aligning with the AdNDP characterization.

**FIGURE 6:**
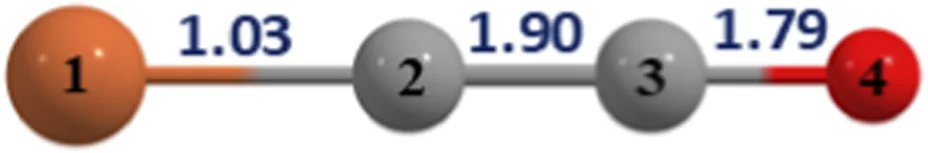
The WBI (in blue) of q1 obtained at the UωB97XD/6–311++G (2d,2p) level.

The bonding properties are further analyzed using WBI. Figure 6 displays the WBIs of all the bonds of q1 determined at the UωB97XD/6–311++G (2d,2p) level of theory. The WBI value of 1.03 between Fe and C2 indicates a single covalent bond between them. The quintet state of the molecule, characterized by a high–spin iron center, contributes fewer electrons to π bonding, hence constraining Fe→ligand backbonding and leading to a predominance of σ Fe–C interactions, which supports a WBI of approximately 1.03. This bonding pattern can be favorable in ISM, where the molecules must be stable with negligible electronic support from the metal. The C–C–O section develops a conjugated π system with enhanced localization of light atoms owing to reduced metal participation. The C2–C3 and C3–O bonds have WBI values of 1.90 and 1.79, respectively, indicating double bonds.

Additional analyses, including NAC, ELF, AIM, and the Laplacian of the electron density, exhibit consistent trends and are hence not elaborated here; results are available in the Supplementary Figures S13, S14.

### 3.5 Infrared (IR) and microwave spectroscopic parameters

**FIGURE 7:**
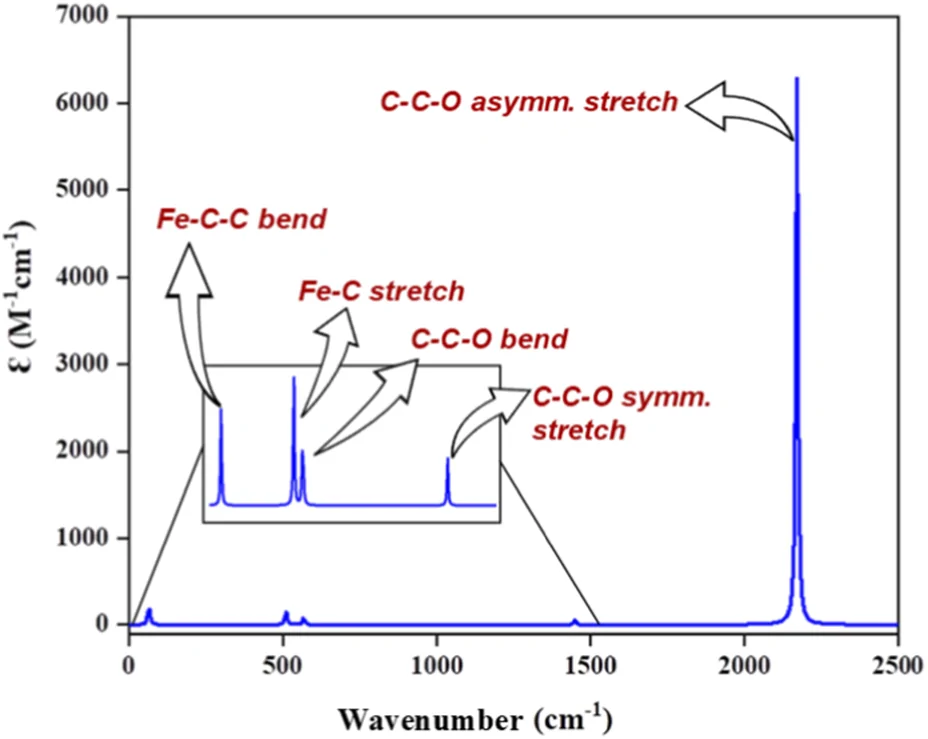
The IR vibrational spectrum of q1 obtained at the UωB97XD/6–311++G (2d,2p) level.

To identify molecules in the laboratory and, by extension, in the ISM, the most significant evidence lies in spectroscopic parameters. Figure 7 illustrates the IR vibrational spectrum of q1 obtained at the UωB97XD/6–311++G (2d,2p) level of theory. The harmonic vibrational frequency analysis of the optimized quintet global minimum of FeC_2_O showed no imaginary frequencies, assuring that the structure represents a true minimum on the PES. The spectrum shows two sets of doubly degenerate π-type bending modes at 63.05 and 566.78 cm^-1^, arising from vibrations perpendicular to the molecular axis in two orthogonal planes, in accordance with the linear geometry of the molecule. The moderate-frequency mode at 508.50 cm^-1^ corresponds to Fe–C stretching. The medium intensity peak at 1448.89 cm^-1^ corresponds to symmetric stretching of the C–C–O fragment, which involves motion of the carbon atom bound to Fe and the terminal oxygen. The highest-frequency mode at 2169.66 cm^-1^ primarily corresponds to the asymmetric stretching of the C–C–O fragment. It results in the most prominent band in the IR spectrum, with remarkably high IR intensity (1832.60 km mol^-1^), establishing it as a distinct spectroscopic signature that may aid experimental detection. The elevated frequency of this mode, relative to standard carbonyl compounds, signifies diminished Fe→CO π back–donation in the high–spin quintet state. The harmonic vibrational frequencies expressed in terms of wavenumber (cm^-1^) and their corresponding IR intensities (km mol^-1^) of q1 computed at the UωB97XD/6–311++G (2d,2p) level are listed in Table 3. All vibrational frequencies reported in this study are harmonic. The IR spectrum suggests a bonding pattern with a strong C_2_O fragment and a weaker Fe–C bond, consistent with analyses of their electronic structures. Moreover, the identification of similar structures in the actual laboratory can be aided by the computationally predicted IR spectra.

**TABLE 3:** Harmonic vibrational frequencies in terms of wavenumber (cm^-1^) and corresponding IR intensities (km mol^-1^) of q1 computed at the UωB97XD/6–311++G (2d,2p) level.

Mode	Wavenumber (cm^-1^)	IR intensity (km mol^-1^)	Assignment
ν_1_	63.05	31.5897	Bending of Fe-C-C framework (degenerate)
ν_2_	63.05	31.5897	Bending of Fe-C-C framework (degenerate)
ν_3_	508.50	47.1025	Fe–C stretching
ν_4_	566.78	12.4840	Bending of C-C-O framework (degenerate)
ν_5_	566.78	12.4840	Bending of C-C-O framework (degenerate)
ν_6_	1448.89	16.4866	Symmetric stretch of C–C–O fragment
ν_7_	2169.66	1832.5953	Asymmetric stretch of C–C–O fragment

Rotational constants are handy astronomical tools because they help discover and study specific molecules in celestial bodies or analyze planetary atmosphere spectra. It is necessary to understand the components of the dipole moment to identify molecules in astronomy, study interstellar chemistry, and analyze planetary atmospheres. Centrifugal distortion constants are also essential for astronomical identification, as they enable accurate spectral predictions, distinguish among isotopic variants, and yield insights into molecular structure and dynamics. Knowing all the parameters discussed above can help enhance our understanding of the chemical composition and evolution of celestial objects. From an experimental point of view, only true minima on the PES are expected to be detectable. Consequently, rotational constants are reported only for the structures that display no imaginary frequencies. Rotational constants for saddle points have been excluded because they lack direct experimental significance. The rotational constants reported in this study are equilibrium (B_e_) values obtained from optimized geometries and do not include vibrational averaging corrections. Table 4 provides the rotational constants and quartic centrifugal distortion constants (τ) for q1 and q7, and Table 5 provides the rotational constants and quartic centrifugal distortion constants (D) for q2, q3, q6, and q8 determined at the UωB97XD/6–311++G (2d,2p) level of theory. The differences in the reported forms are ascribed to the alterations in the mathematical representation of the quartic distortion constants, rather than to any actual deviations in rotational behavior. It is observed that precise spectroscopic predictions for transition-metal systems may necessitate explicit consideration of core–valence correlation at the wavefunction level (Drosou et al., 2022; Xu et al., 2015). Such considerations exceed the parameters of the current exploratory study.

**TABLE 4:** Inertial axis dipole moment components, absolute dipole moments (in Debye), centrifugal distortion constants τ (in MHz), and equilibrium rotational constants (in MHz) of the optimized structures FeC_2_O in their corresponding quintet electronic states derived from harmonic frequency calculations computed at the UωB97XD/6–311++G (2d,2p) level of theory.

Isomer	μ_a_	μ_b_	μ_c_	|μ|	τ_a_	τ_b_	τ_c_	D_e_	A_e_	B_e_	C_e_
q1	8.52	0	0	8.52	0	0	−4.39x10^−4^	1.09x10^−4^	0	1881.62	1881.63
q7	13.44	0	0	13.44	0	0	−6.61x10^−4^	1.65x10^−4^	0	2151.18	2151.18

**TABLE 5:** Inertial axis dipole moment components, absolute dipole moments (in Debye), centrifugal distortion constants D (in MHz), and equilibrium rotational constants (in MHz) of the optimized structures FeC_2_O in their corresponding quintet electronic states derived from harmonic frequency calculations computed at the UωB97XD/6–311++G (2d,2p) level of theory.

Isomer	μ_a_	μ_b_	μ_c_	|μ|	D_J_	D_K_	D_JK_	d_1_	d_2_	A_e_	B_e_	C_e_
q2	1.03	0	0	1.03	1.03x10^−3^	3.72x10^−2^	1.58x10^−1^	−6.71x10^−5^	−8.16x10^−2^	53,008.19	3594.06	3365.85
q3	−0.46	−3.60	0	3.63	4.48x10^−3^	3.23x10^–−1^	−2.94x10^−2^	8.79x10^−4^	3.79x10^−2^	20,293.17	5133.55	4869.89
q6	8.26	−1.11	0	8.34	6.53x10^−4^	7.95x10^−1^	6.62x10^−2^	−3.09x10^−5^	−2.80x10^−2^	34,399.31	2756.84	2552.29
q8	2.70	−0.69	0	2.79	3.84x10^−3^	3.98x10^0^	2.43x10^−1^	−1.86x10^−4^	−9.99x10^−2^	52,354.87	3562.43	3335.47

### 3.6 Density of states (DOS)

**FIGURE 8:**
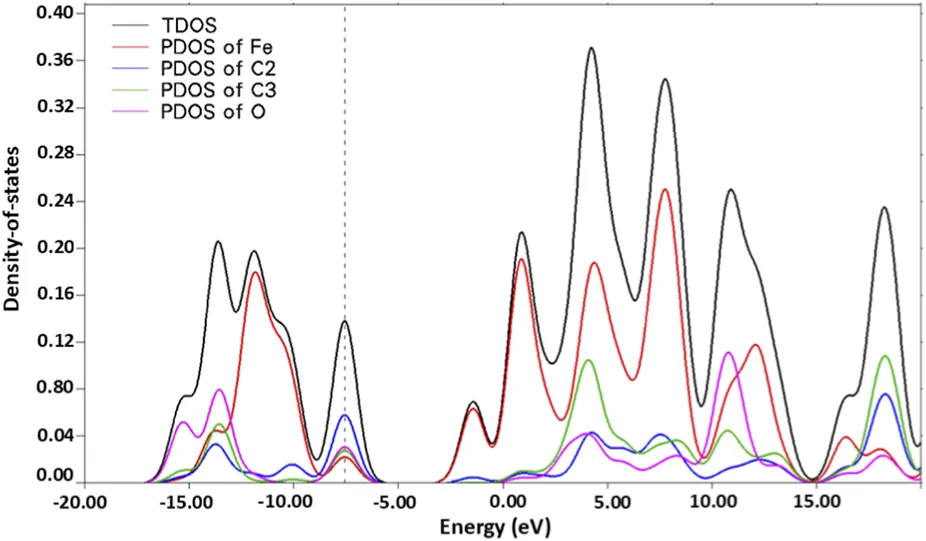
The density of states plot of q1 obtained at the UωB97XD/6–311++G (2d,2p) level.

A DOS plot graphically displays the number of energy states accessible for electrons in a system at various energy levels. The total density of states (TDOS) represents the overall distribution of energy states within the system. The partial density of states (PDOS) signifies the contributions from distinct parts of the molecule or system to the overall DOS. Figure 8 shows the DOS plot for q1. The dashed line indicates the position of the HOMO. Peaks in the DOS correspond to high concentrations of energy levels at that energy, whereas troughs represent regions with a reduced number of available energy states. In Figure 8, the black curve represents TDOS. In addition, the red, blue, green, and pink curves represent the contributions of Fe, C2, C3, and O atoms, respectively. The TDOS shows an evident energy gap near the Fermi level, establishing the molecule’s electronic stability. An orbital composition analysis was conducted with NAO-based orbital decomposition to quantitatively analyze the frontier electronic structure of q1. The spin-resolved atomic composition (%) of the frontier molecular orbitals of q1, obtained at the UωB97XD/6–311++G (2d,2p) level of theory, is shown in Supplementary Table S18 in the Supplementary Material. It indicates that the α-HOMO and β-HOMO are dominated by contributions from the C_2_O fragment, whereas α-LUMO and β-LUMO are mainly localized on the Fe atom, contributing 86.3% and 94.1%, respectively. This further validates the electronic structure obtained from orbital and charge analysis.

### 3.7 Astrochemical implications

While the present study focuses on the intrinsic electronic structure and spectroscopic properties of FeC_2_O, the possibility of its formation under ISM conditions warrants further investigation. A potential pathway entails the sequential synthesis commencing with Fe and CO to produce FeCO, followed by a reaction with atomic carbon to generate FeC_2_O. Alternatively, considering that FeC radical has been identified in space (Koelemay and Ziurys, 2023), insertion reactions such as FeC + CO → FeC_2_O constitute a chemically plausible pathway. A third hypothesis pertains to the interaction of atomic Fe with transitory C_2_O (ketenylidene) fragments generated by the active processing of ice grain mantles that are rich in CO (Ohishi, et al., 1991; Urso et al., 2019). Although these paths are hypothetical, the synthesis of FeC_2_O in ISM is expected to happen through gas-phase reactions involving small iron-containing entities, rather than by the direct modification of pure iron grains, as pure iron grains are considered rare there (Kimura et al., 2017). The low gas-phase abundance of iron and the strong reactivity of C_2_O imply that FeC_2_O is probably a transient or low-abundance species rather than a significant molecular reservoir. It indicates that although the efficient synthesis of FeC_2_O is not established, there is potential for its formation under specific conditions, such as intermittent iron release from dust grains in shocked areas or in circumstellar envelopes, making it chemically plausible and spectroscopically identifiable. A quantitative evaluation of reaction energetics and kinetics is beyond the scope of the present study and will be carried out in the future.

The intrinsic electronic stability of FeC_2_O in isolation is indicated by the studies reported in the present work; however, these studies do not assess its ability to survive in the ISM, where it is exposed to hazards such as cosmic rays and UV radiation. These factors have the potential to induce electronic excitation or photodissociation (Heays et al., 2017; Schippers and Müller, 2020) which implies that FeC_2_O is unlikely to be abundant or long-lived in space. Under interstellar conditions, FeC_2_O is anticipated to be a transient or low-abundance species, with detection limited to shielded areas or environments with recent energetic activity.

The non-detection of FeC_2_O in ISM does not preclude its transient production. In contrast to FeC and FeCN, which possess efficient formation pathways involving carbon- and nitrogen-containing radicals, the production of FeC_2_O may be impeded by the low quantity and high reactivity of the C_2_O fragment. The absence of laboratory rotational spectra for neutral FeC_2_O has certainly impeded astronomical searches. Molecules without laboratory rotational spectra are infrequently investigated in astronomical surveys, potentially leading to non-detection despite their transient presence.

## 4 Conclusion

The isomers of FeC_2_O were investigated using two density functional methods with and without an effective core potential, with emphasis on the lowest-energy quintet electronic state. The linear quintet structure q1 is the lowest energy FeC_2_O isomer within the DFT framework considered in the study, and is identified to be the global minimum at the UωB97XD/6–311++G (2d,2p) level of theory. The chemical bonding characteristics of the global minimum of FeC_2_O are further investigated at the UωB97XD level with the 6–311++G (2d,2p) basis set. Various quantum-chemical tools were employed to elucidate the nature of chemical bonding and structural properties, including AdNDP, MO analysis, and WBI. These investigations indicate primarily localized Fe–C and C–O bonds, poor Fe–ligand covalency, and a robustly bonded C_2_O fragment.

The computed IR spectrum, along with a significant HOMO–LUMO gap, suggests increased electronic stability of q1, while the DOS and molecular orbital analysis indicate substantial Fe 3d contributions to the frontier electronic structure and moderate metal–ligand orbital interactions. Dissociation-energy calculations conducted reveal that the Fe + C_2_O and FeC + CO fragmentation channels are highly endothermic, while the Fe–C and C–C scans show a continuous increase in energy with bond elongation. These findings further establish that q1 as a strongly bound minimum and further substantiate its energetic stability.

Single-point (U)CCSD(T) computations and T_1_ diagnostic values further reveal that the FeC_2_O PES is sensitive to electron correlation, and certain singlet, triplet, and quintet structures exhibit substantial multireference character. Additional UCCSD and CASSCF optimizations of quintet isomers confirm that q1 remains the lowest-energy quintet structure, further supporting the assignment of q1 as the preferred low-energy FeC_2_O candidate within the computational framework employed.

The unique spectroscopic fingerprints and electronic structure of FeC_2_O make it a viable target for future astrochemical detection, supported by computational studies of iron–carbon–oxygen molecules. The structural and energy parameters, together with the infrared and microwave spectroscopic parameters, including rotational and centrifugal distortion constants, reported in the present work, can substantially assist the experimentalists in identifying this molecule in the ISM.

## Data Availability

The original contributions presented in the study are included in the article/Supplementary Material, further inquiries can be directed to the corresponding author.
