# Supplementary material for: Computational perspective on structure, energetics, and bonding of FeC2O isomers with astrochemical significance
Source: Front Chem. 2026 Aug 14;14:1874752. doi: 10.3389/fchem.2026.1874752 (PMC13522136; doi:10.3389/fchem.2026.1874752)
Supplement: Supplementary file 1 [file Supplementaryfile1.docx]

Supplementary Material for:

**Computational Perspective on Structure, Energetics, and Bonding of FeC_2_O isomers with Astrochemical Significance**

**Abhirami R J and Senthur Pandi R^*^**

Department of Physics, School of Advanced Sciences, Vellore Institute of Technology, Vellore 632014, Tamil Nadu, India.

Corresponding author email: senthur.pandi@vit.ac.in

**Contents**

List of Figures (S1 to S14)

List of Tables (S1 to S21)

**List of Figures (Revised / newly added figures are highlighted)**

| S1 | Isomers of FeC_2_O with non-zero imaginary frequencies in their corresponding quintet electronic states, with their point group, ZPVE corrected relative energies (in kcal mol^–1^), dipole moment (in Debye), and number of imaginary frequencies (Nimag) computed at the UωB97XD/6–311++G(2d,2p) theoretical level. | S6 |
| --- | --- | --- |
| S2 | Isomer of FeC_2_O (q11) in its quintet electronic state obtained with the UTPSSh/6-311++G(2d,2p), UTPSSh/SDD & 6-311++G(2d,2p) and UωB97XD/SDD & 6-311++G(2d,2p) theoretical levels. | S6 |
| S3 | Isomers of FeC_2_O (q12 and q13) in its quintet electronic state obtained with the UTPSSh/6-311++G(2d,2p), and UTPSSh/SDD & 6-311++G(2d,2p) theoretical levels. | S6 |
| S4 | Isomer of FeC_2_O (q14) in their quintet electronic state obtained only with the UTPSSh/SDD & 6-311++G(2d,2p) theoretical level. | S6 |
| S5 | Isomers of FeC_2_O in their corresponding triplet electronic states, with their point group, ZPVE corrected relative energies (in kcal mol^–1^), dipole moment (in Debye), and number of imaginary frequencies (Nimag) computed at the UωB97XD/6-311++G(2d,2p) theoretical level. | S7 |
| S6 | Isomers of FeC_2_O in their corresponding singlet electronic states, with their point group, ZPVE corrected relative energies (in kcal mol^–1^), dipole moment (in Debye), and number of imaginary frequencies (Nimag) computed at the ωB97XD/6–311++G(2d,2p) level of theory. | S7 |
| S7 | Isomer of FeC_2_O (s17) in its corresponding singlet electronic state obtained with the TPSSh/6-311++G(2d,2p), TPSSh/SDD & 6-311++G(2d,2p) and ωB97XD/SDD & 6-311++G(2d,2p) theoretical levels. | S8 |
| S8 | Isomer of FeC_2_O (s18) in its corresponding singlet electronic state obtained with the TPSSh/6-311++G(2d,2p), and TPSSh/SDD & 6-311++G(2d,2p) theoretical levels. | S8 |
| S9 | Isomers of FeC_2_O (s19 and s20) in their corresponding singlet electronic state obtained only with the TPSSh/6-311++G(2d,2p) theoretical level. | S8 |
| S10 | s1, t2(s1), and q1(s3) in their singlet, triplet, and quintet electronic states, respectively, with the point groups, relative energies (in kcal mol^-1^) calculated at CCSD(T)/SDD&6-311++g(2d,2p)//(U)ωB97XD/SDD&6-311++g(2d,2p) level of theory. | S8 |
| S11 | Isomers of FeC_2_O in its quintet electronic state obtained with the CCSD/6-311++G(2d,2p) level of theory. | S9 |
| S12 | The β-spin AdNDP orbitals of q1 computed at the UωB97XD/6–311++G(2d,2p) level. | S9 |
| S13 | The NACs of all atoms of q1 computed at the UωB97XD/6–311++G(2d,2p) level. | S9 |
| S14 | At the UωB97XD/6–311++G(2d,2p) level, (a) the color–filled map of ELF and (b) the contour map of the Laplacian of the electron density generated for q1. | S9 |

**List of Tables**

| S1 | Isomers of FeC_2_O in their corresponding quintet state computed at the UTPSSh/6-311++G(2d,2p) theoretical level with point group, total energy (in a.u)**,** zero-point correction (in a.u), ZPVE corrected total energy (E+ZPVE; in a.u), relative energy with ZPVE corrections (ΔE + ZPVE; in a.u), relative energies with ZPVE corrections (ΔE + ZPVE) in kcal mol^-1^, absolute dipole moment (in Debye), number of imaginary frequencies (NImag), expectation value of the total spin after spin annihilation, <S^2^> and %error (Δ). | S10 |
| --- | --- | --- |
| S2 | Isomers of FeC_2_O in their corresponding quintet state computed at the UTPSSh/SDD & 6-311++G(2d,2p) theoretical level with point group, total energy (in a.u)**,** zero-point correction (in a.u), ZPVE corrected total energy (E+ZPVE; in a.u), relative energy with ZPVE corrections (ΔE + ZPVE; in a.u), relative energies with ZPVE corrections (ΔE + ZPVE) in kcal mol^-1^, absolute dipole moment (in Debye), number of imaginary frequencies (NImag), expectation value of the total spin after spin annihilation, <S^2^> and %error (Δ). | S11 |
| S3 | Isomers of FeC_2_O in their corresponding quintet state computed at the UωB97XD/6-311++G(2d,2p) theoretical level with point group, total energy (in a.u)**,** zero-point correction (in a.u), ZPVE corrected total energy (E+ZPVE; in a.u), relative energy with ZPVE corrections (ΔE + ZPVE; in a.u), relative energies with ZPVE corrections (ΔE + ZPVE) in kcal mol^-1^, absolute dipole moment (in Debye), number of imaginary frequencies (NImag), expectation value of the total spin after spin annihilation, <S^2^> and %error (Δ). | S12 |
| S4 | Isomers of FeC_2_O in their corresponding quintet state computed at the UωB97XD/ SDD & 6-311++G(2d,2p) theoretical level with point group, total energy (in a.u)**,** zero-point correction (in a.u), ZPVE corrected total energy (E+ZPVE; in a.u), relative energy with ZPVE corrections (ΔE + ZPVE; in a.u), relative energies with ZPVE corrections (ΔE + ZPVE) in kcal mol^-1^, absolute dipole moment (in Debye), number of imaginary frequencies (NImag), expectation value of the total spin after spin annihilation, <S^2^> and %error (Δ). | S12 |
| S5 | Isomers of FeC_2_O in their corresponding triplet state computed at the UωB97XD/ 6-311++G(2d,2p) theoretical level with point group, total energy (in a.u)**,** zero-point correction (in a.u), ZPVE corrected total energy (E+ZPVE; in a.u), relative energy with ZPVE corrections (ΔE + ZPVE; in a.u), relative energies with ZPVE corrections (ΔE + ZPVE) in kcal mol^-1^, absolute dipole moment (in Debye), number of imaginary frequencies (NImag), expectation value of the total spin after spin annihilation, <S^2^> and %error(Δ). | S13 |
| S6 | Isomers of FeC_2_O in their corresponding singlet state computed at the TPSSh/6-311++G(2d,2p) theoretical level with point group, total energy (in a.u)**,** zero-point correction (in a.u), ZPVE corrected total energy (E+ZPVE; in a.u), relative energy with ZPVE corrections (ΔE + ZPVE; in a.u), relative energies with ZPVE corrections (ΔE + ZPVE) in kcal mol^-1^, absolute dipole moment (in Debye), and the number of imaginary frequencies (NImag). | S14 |
| S7 | Isomers of FeC_2_O in their corresponding singlet state computed at the TPSSh/SDD & 6-311++G(2d,2p) theoretical level with point group, total energy (in a.u)**,** zero-point correction (in a.u), ZPVE corrected total energy (E+ZPVE; in a.u), relative energy with ZPVE corrections (ΔE + ZPVE; in a.u), relative energies with ZPVE corrections (ΔE + ZPVE) in kcal mol^-1^, absolute dipole moment (in Debye), and the number of imaginary frequencies (NImag). | S15 |
| S8 | Isomers of FeC_2_O in their corresponding singlet state computed at the ωB97XD/6-311++G(2d,2p) theoretical level with point group, total energy (in a.u)**,** zero-point correction (in a.u), ZPVE corrected total energy (E+ZPVE; in a.u), relative energy with ZPVE corrections (ΔE + ZPVE; in a.u), relative energies with ZPVE corrections (ΔE + ZPVE) in kcal mol^-1^, absolute dipole moment (in Debye), and the number of imaginary frequencies (NImag). | S16 |
| S9 | Isomers of FeC_2_O in their corresponding singlet state computed at the ωB97XD/ SDD & 6-311++G(2d,2p) theoretical level with point group, total energy (in a.u)**,** zero-point correction (in a.u), ZPVE corrected total energy (E+ZPVE; in a.u), relative energy with ZPVE corrections (ΔE + ZPVE; in a.u), relative energies with ZPVE corrections (ΔE + ZPVE) in kcal mol^-1^, absolute dipole moment (in Debye), and the number of imaginary frequencies (NImag). | S17 |
| S10 | ZPVE corrected relative energies of FeC_2_O isomers (in kcal mol^–1^) in their corresponding singlet electronic states obtained at different levels. | S18 |
| S11 | Optimized geometrical parameters (bond lengths in Å and bond angles in Degrees) of FeC_2_O isomers in their corresponding quintet states, calculated at different levels of theory. | S19 |
| S12 | Optimized geometrical parameters (bond lengths in Å and bond angles in Degrees) of FeC_2_O isomers in their corresponding singlet states, calculated at different levels of theory. | S22 |
| S13 | Comparison of the relative energies (ΔE + ZPVE) of FeC_2_O isomers in their quintet electronic state optimized at UωB97XD functional with 6-311++G(2d,2p) basis set, and single-point energy calculation at the CCSD(T) with 6-311++G(2d,2p) basis set. | S26 |
| S14 | Comparison of the relative energies (ΔE + ZPVE) of FeC_2_O isomers in their triplet electronic state optimized at UωB97XD functional with 6-311++G(2d,2p) basis sets, and single-point energy calculation at the CCSD(T) with 6-311++G(2d,2p) basis sets. | S26 |
| S15 | Comparison of the relative energies (ΔE + ZPVE) of FeC_2_O isomers in their singlet electronic state optimized at ωB97XD functional with 6-311++G(2d,2p) basis set, and single-point energy calculation at the CCSD(T) with 6-311++G(2d,2p) basis set. | S27 |
| S16 | Comparison of the relative energies (ΔE + ZPVE) of FeC_2_O isomers in their quintet electronic state optimized at UωB97XD/6-311++G(2d,2p), and CCSD/6-311++G(2d,2p) levels of theory. | S27 |
| S17 | Comparison of the relative energies (ΔE + ZPVE) of FeC_2_O isomers in their quintet electronic state optimized at UωB97XD/6-311++G(2d,2p), and CASSCF/def2-tzvp levels of theory. | S28 |
| S18 | Spin-resolved atomic composition (%) of the frontier molecular orbitals of q1 at the UωB97XD/6–311++G(2d,2p) level. | S28 |
| S19 | The Cartesian coordinates (in Angstrom units) of isomers of FeC_2_O in their quintet electronic state obtained at different levels of theory. | S29 |
| S20 | The Cartesian coordinates (in Angstrom units) of isomers of FeC_2_O in their triplet electronic state obtained at the UωB97XD/6-311++G(2d,2p) theoretical level. | S33 |
| S21 | The Cartesian coordinates (in Angstrom units) of isomers of FeC_2_O in their singlet electronic state obtained at different levels of theory. | S34 |

**Figure S1** Isomers of FeC_2_O with non-zero imaginary frequencies in their corresponding quintet electronic states, with their point group, ZPVE corrected relative energies (in kcal mol^–1^), dipole moment (in Debye), and number of imaginary frequencies (Nimag) obtained at the UωB97XD/6-311++G(2d,2p) theoretical level. The corresponding singlet states are included in parentheses

**Figure S2** Isomer of FeC_2_O (q11) in its quintet electronic state obtained with the UTPSSh/6-311++G(2d,2p), UTPSSh/SDD & 6-311++G(2d,2p) and UωB97XD/SDD & 6-311++G(2d,2p) theoretical levels.

**Figure S3** Isomers of FeC_2_O (q12 and q13) in its quintet electronic state obtained with the UTPSSh/6-311++G(2d,2p), and UTPSSh/SDD & 6-311++G(2d,2p) theoretical levels.

**Figure S4** Isomer of FeC_2_O (q14) in its quintet electronic state obtained only with the UTPSSh/SDD & 6-311++G(2d,2p) theoretical level.

**Figure S5** Isomers of FeC_2_O in their corresponding triplet electronic states, with their point group, ZPVE corrected relative energies (in kcal mol^–1^), dipole moment (in Debye), and number of imaginary frequencies (Nimag) obtained at the UωB97XD/6-311++G(2d,2p) theoretical level. The corresponding singlet states are included in parentheses.

**Figure S6** Isomers of FeC_2_O in their corresponding singlet electronic states, with their point group, ZPVE corrected relative energies (in kcal mol^–1^), dipole moment (in Debye), and number of imaginary frequencies (Nimag) computed at the ωB97XD/6–311++G(2d,2p) level of theory.

**Figure S7** Isomer of FeC_2_O (s17) in its singlet electronic state obtained with the TPSSh/6-311++G(2d,2p), TPSSh/SDD & 6-311++G(2d,2p) and ωB97XD/SDD & 6-311++G(2d,2p) theoretical levels.

**Figure S8** Isomer of FeC_2_O (s18) in its singlet electronic state obtained with the TPSSh/6-311++G(2d,2p), and TPSSh/SDD & 6-311++G(2d,2p) theoretical levels.

**Figure S9** Isomers of FeC_2_O (s19 and s20) in their singlet electronic state obtained only with the TPSSh/6- 311++G(2d,2p) theoretical level.

**Figure S10** s1, t2(s1), and q1(s3) in their singlet, triplet, and quintet electronic states, respectively, with the point groups, relative energies (in kcal mol^-1^) calculated at CCSD(T)/SDD&6-311++G(2d,2p) //(U)ωB97XD/SDD&6-311++G(2d,2p) level of theory.

**Figure S11** Isomers of FeC_2_O in its quintet electronic state obtained with the CCSD/6-311++G(2d,2p) level of theory.

**Figure S12** The β-spin AdNDP bonding patterns of q1 with occupation numbers (ONs) obtained at the UωB97XD/6–311++G(2d,2p) level.

**Figure S13** The NACs of all atoms of q1 computed at the UωB97XD/6–311++G(2d,2p) level.

**Figure S14** At the UωB97XD/6–311++G(2d,2p) level, (a) the color–filled map of ELF and (b) the contour map of the Laplacian of the electron density generated for q1

**Table S1** Point group, total energy (in a.u)**,** zero-point correction (in a.u), ZPVE corrected total energy (E+ZPVE; in a.u), relative energy with ZPVE corrections (ΔE + ZPVE; in a.u), relative energies with ZPVE corrections (ΔE + ZPVE) in kcal mol^-1^, absolute dipole moment (in Debye), number of imaginary frequencies (NImag), expectation value of the total spin after spin annihilation, <S^2^> and %error (Δ) of FeC_2_O isomers in their corresponding quintet state computed at the UTPSSh/6-311++G(2d,2p) theoretical level. The corresponding singlet states are included in parentheses.

| **Isomer** | **Point group** | **Energy (a.u.)** | **ZPVE**  **(a.u.)** | **E+ZPVE**  **(a.u.)** | **∆E+ZPVE**  **(a.u)** | **∆E+ZPVE**  **(kcal/mol)** | **μ**  **(Debye)** | **Nimag** | **<S^2^>** | **% error**  **(Δ)** |
| --- | --- | --- | --- | --- | --- | --- | --- | --- | --- | --- |
| q11(s19) | C_s_ | -1415.0739 | 0.011456 | -1415.0625 | 0 | 0 | 6.6117 | 0 | 6.0002 | 0.00 |
| q1(s3) | C_∞v_ | -1415.0734 | 0.011445 | -1415.0619 | 0.000534 | 0.335085 | 7.5448 | 2 | 6.0002 | 0.00 |
| q12 | C_s_ | -1414.9931 | 0.008537 | -1414.9846 | 0.077891 | 48.876603 | 2.7648 | 0 | 6.0053 | 0.08 |
| q3(s9) | C_s_ | -1414.9836 | 0.009294 | -1414.9743 | 0.088150 | 55.314125 | 3.3921 | 0 | 6.0016 | 0.02 |
| q10(s8) | C_∞v_ | -1414.9823 | 0.008487 | -1414.9739 | 0.088637 | 55.619718 | 2.4560 | 1 | 6.0015 | 0.03 |
| q2(s10) | C_1_ | -1414.9743 | 0.008032 | -1414.9663 | 0.096232 | 60.385580 | 0.3888 | 0 | 6.0120 | 0.20 |
| q7(s16) | C_∞v_ | -1414.9681 | 0.009282 | -1414.9589 | 0.103644 | 65.036610 | 11.9536 | 2 | 6.0002 | 0.00 |
| q4(s14) | C_∞v_ | -1414.9617 | 0.007797 | -1414.9539 | 0.108600 | 68.146500 | 0.4585 | 2 | 6.0312 | 0.52 |
| q5(s12) | C_s_ | -1414.9525 | 0.008032 | -1414.9445 | 0.117999 | 74.044373 | 5.7977 | 1 | 6.0001 | 0.00 |
| q9(s11) | C_s_ | -1414.9502 | 0.007224 | -1414.9429 | 0.119517 | 74.996918 | 5.8283 | 0 | 6.0002 | 0.00 |
| q6(s13) | C_s_ | -1414.9503 | 0.008186 | -1414.9422 | 0.120331 | 75.507703 | 7.6394 | 0 | 6.0002 | 0.00 |
| q13(s18) | C_∞v_ | -1414.9044 | 0.008102 | -1414.8963 | 0.166159 | 104.264773 | 6.8820 | 2 | 6.0022 | 0.04 |

**Table S2** Point group, total energy (in a.u)**,** zero-point correction (in a.u), ZPVE corrected total energy (E+ZPVE; in a.u), relative energy with ZPVE corrections (ΔE + ZPVE; in a.u), relative energies with ZPVE corrections (ΔE + ZPVE) in kcal mol^-1^, absolute dipole moment (in Debye), number of imaginary frequencies (NImag), expectation value of the total spin after spin annihilation, <S^2^> and %error(Δ) of FeC_2_O isomers in their corresponding quintet state computed at the UTPSSh/SDD&6-311++G(2d,2p) theoretical level. The corresponding quintet states are included in parentheses.

| **Isomer** | **Point group** | **Energy**  **(a.u.)** | **ZPVE**  **(a.u.)** | **E+ZPVE**  **(a.u.)** | **∆E+ZPVE**  **(a.u)** | **∆E+ZPVE**  **(kcal/mol)** | **μ**  **(Debye)** | **Nimag** | **<S^2^>** | **% error**  **(Δ)** |
| --- | --- | --- | --- | --- | --- | --- | --- | --- | --- | --- |
| q11(s19) | C_s_ | -275.2343 | 0.011417 | -275.2228 | 0 | 0 | 6.1898 | 0 | 6.0003 | 0 |
| q1(s3) | C_∞v_ | -275.2313 | 0.011464 | -275.2199 | 0.002930 | 1.838575 | 7.8743 | 2 | 6.0002 | 0 |
| q12 | C_s_ | -275.1440 | 0.008379 | -275.1357 | 0.087169 | 54.698548 | 2.8621 | 0 | 6.0135 | 0.22 |
| q7(s16) | C_∞v_ | -275.1416 | 0.008886 | -275.1328 | 0.090081 | 56.525828 | 6.5231 | 2 | 6.0066 | 0.11 |
| q10(s8) | C_∞v_ | -275.1340 | 0.008495 | -275.1255 | 0.097373 | 61.101558 | 2.5285 | 0 | 6.0045 | 0.07 |
| q3(s9) | C_1_ | -275.1311 | 0.009106 | -275.1219 | 0.100884 | 63.304710 | 3.3954 | 0 | 6.0030 | 0.05 |
| q2(s10) | C_s_ | -275.1298 | 0.007961 | -275.1218 | 0.101015 | 63.386913 | 0.6893 | 0 | 6.0301 | 0.50 |
| q6(s13) | C_s_ | -275.1188 | 0.008196 | -275.1106 | 0.112245 | 70.433738 | 7.4490 | 0 | 6.0001 | 0 |
| q4(s14) | C_∞v_ | -275.1109 | 0.008186 | -275.1027 | 0.120098 | 75.361495 | 1.6378 | 0 | 6.0268 | 0.45 |
| q9(s11) | C_s_ | -275.1076 | 0.006800 | -275.1008 | 0.122007 | 76.559393 | 5.5640 | 1 | 6.0003 | 0 |
| q5(s12) | C_s_ | -275.1062 | 0.015640 | -275.0906 | 0.132265 | 82.996288 | 6.0024 | 0 | 6.0001 | 0 |
| q13(q18) | C_∞v_ | -275.0582 | 0.008166 | -275.0500 | 0.172839 | 108.456473 | 7.1131 | 2 | 6.0029 | 0.05 |
| q15 | C_2v_ | -275.0476 | 0.005866 | -275.0417 | 0.181135 | 113.662213 | 3.8424 | 1 | 6.0150 | 0.25 |

**Table S3** Point group, total energy (in a.u)**,** zero-point correction (in a.u), ZPVE corrected total energy (E+ZPVE; in a.u), relative energy with ZPVE corrections (ΔE + ZPVE; in a.u), relative energies with ZPVE corrections (ΔE + ZPVE) in kcal mol^-1^, absolute dipole moment (in Debye), number of imaginary frequencies (NImag), expectation value of the total spin after spin annihilation, <S^2^> and %error(Δ) of FeC_2_O isomers in their corresponding quintet state computed at the UωB97XD/6-311++G(2d,2p) theoretical level. The corresponding quintet states are included in parentheses.

| **Isomer** | **Point**  **group** | **Energy**  **(a.u.)** | **ZPVE**  **(a.u.)** | **E+ZPVE**  **(a.u.)** | **∆E+ZPVE**  **(a.u)** | **∆E+ZPVE**  **(kcal/mol)** | **μ**  **(Debye)** | **Nimag** | **<S^2^>** | **%error**  **(Δ)** |
| --- | --- | --- | --- | --- | --- | --- | --- | --- | --- | --- |
| q1(s3) | C_∞v_ | -1414.9919 | 0.012117 | -1414.9797 | 0 | 0 | 8.5205 | 0 | 6.0005 | 0.01 |
| q2(s10) | C_2v_ | -1414.8809 | 0.007534 | -1414.8734 | 0.106297 | 66.701367 | 0.5363 | 0 | 6.0275 | 0.46 |
| q3(s9) | C_1_ | -1414.8807 | 0.009205 | -1414.8715 | 0.108168 | 67.875420 | 2.7822 | 0 | 6.0029 | 0.05 |
| q4(s14) | C_∞v_ | -1414.8768 | 0.008168 | -1414.8687 | 0.111018 | 69.663795 | 0.9109 | 2 | 6.0387 | 0.64 |
| q5(s12) | C_s_ | -1414.8697 | 0.008447 | -1414.8613 | 0.118398 | 74.294745 | 5.6622 | 1 | 6.0001 | 0.00 |
| q6(s13) | C_s_ | -1414.8694 | 0.008560 | -1414.8608 | 0.118850 | 74.578375 | 7.1796 | 0 | 6.0001 | 0.00 |
| q7(s16) | C_∞v_ | -1414.8692 | 0.010611 | -1414.8586 | 0.121084 | 75.980210 | 13.2517 | 0 | 6.0005 | 0.01 |
| q8(s7) | C_s_ | -1414.8642 | 0.007692 | -1414.8565 | 0.123232 | 77.328080 | 0.7224 | 0 | 6.0025 | 0.04 |
| q9(s11) | C_s_ | -1414.8615 | 0.007237 | -1414.8543 | 0.125433 | 78.709207 | 5.8364 | 1 | 6.0001 | 0.00 |
| q10(s8) | C_∞v_ | -1414.8522 | 0.008722 | -1414.8435 | 0.136227 | 85.482442 | 1.6562 | 2 | 6.0094 | 0.16 |

**Table S4** Point group, total energy (in a.u)**,** zero-point correction (in a.u), ZPVE corrected total energy (E+ZPVE; in a.u), relative energy with ZPVE corrections (ΔE + ZPVE; in a.u), relative energies with ZPVE corrections (ΔE + ZPVE) in kcal mol^-1^, absolute dipole moment (in Debye), number of imaginary frequencies (NImag), expectation value of the total spin after spin annihilation, <S^2^> and %error(Δ) of FeC_2_O isomers in their corresponding quintet state computed at the UωB97XD/ SDD & 6-311++G(2d,2p) theoretical level. The corresponding quintet states are included in parentheses.

| **Isomer** | **Point**  **group** | **Energy**  **(a.u.)** | **ZPVE**  **(a.u.)** | **E+ZPVE**  **(a.u.)** | **∆E+ZPVE**  **(a.u)** | **∆E+ZPVE**  **(kcal/mol)** | **μ**  **(Debye)** | **Nimag** | **<S^2^>** | **% error**  **(Δ)** |
| --- | --- | --- | --- | --- | --- | --- | --- | --- | --- | --- |
| q1(s3) | C_∞v_ | -275.2042 | 0.012366 | -275.1919 | 0 | 0 | 8.7453 | 0 | 6.0005 | 0 |
| q11(s19) | C_s_ | -275.1956 | 0.011640 | -275.1840 | 0.007850 | 4.925875 | 6.8732 | 0 | 6.0003 | 0 |
| q10(s8) | C_∞v_ | -275.0902 | 0.009715 | -275.0804 | 0.111414 | 69.912285 | 2.9113 | 0 | 6.0166 | 0.28 |
| q4(s14) | C_∞v_ | -275.0860 | 0.008190 | -275.0779 | 0.113999 | 71.534372 | 0.6298 | 2 | 6.0423 | 0.71 |
| q6(s13) | C_s_ | -275.0835 | 0.008821 | -275.0747 | 0.117161 | 73.518527 | 7.546 | 0 | 6.0005 | 0 |
| q7(s16) | C_∞v_ | -275.0829 | 0.010844 | -275.0721 | 0.119795 | 75.171362 | 5.6236 | 0 | 6.04 | 0.67 |
| q2(s10) | C_1_ | -275.0732 | 0.007526 | -275.0657 | 0.126175 | 79.174812 | 0.2063 | 0 | 6.0244 | 0.41 |
| q8(s7) | C_s_ | -275.0702 | 0.007585 | -275.0626 | 0.129278 | 81.121945 | 3.1675 | 0 | 6.0044 | 0.07 |
| q5(s12) | C_s_ | -275.0534 | 0.008310 | -275.0450 | 0.146817 | 92.127667 | 6.2794 | 1 | 6.0002 | 0 |

**Table S5** Point group, total energy (in a.u)**,** zero-point correction (in a.u), ZPVE corrected total energy (E+ZPVE; in a.u), relative energy with ZPVE corrections (ΔE + ZPVE; in a.u), relative energies with ZPVE corrections (ΔE + ZPVE) in kcal mol^-1^, absolute dipole moment (in Debye), number of imaginary frequencies (NImag), expectation value of the total spin after spin annihilation, <S^2^> and %error(Δ) of FeC_2_O isomers in their corresponding triplet state computed at the UωB97XD/6-311++G(2d,2p) theoretical level. The corresponding singlet states are included in parentheses.

| **Isomer** | **Point group** | **Energy (a.u.)** | **ZPVE (a.u.)** | **E+ZPVE (a.u.)** | **∆E+ZPVE (a.u)** | **∆E+ZPVE (kcal/mol)** | **μ (Debye)** | **Nimag** | **<S^2^>** | **%error**  **(Δ)** |
| --- | --- | --- | --- | --- | --- | --- | --- | --- | --- | --- |
| t1(s3) | C_∞v_ | -1414.9855 | 0.011747 | -1414.9737 | 0 | 0 | 5.5158 | 0 | 2.2679 | 13.39 |
| t2(s1) | C_s_ | -1414.9318 | 0.009482 | -1414.9223 | 0.051409 | 32.259148 | 2.7220 | 0 | 2.0720 | 3.60 |
| t3(s4) | C_s_ | -1414.9290 | 0.010997 | -1414.9180 | 0.055702 | 34.953005 | 4.7532 | 0 | 2.0413 | 2.06 |
| t4(s16) | C_∞v_ | -1414.9061 | 0.010071 | -1414.8961 | 0.077644 | 48.721610 | 9.0744 | 2 | 2.6440 | 32.20 |
| t5(s12) | C_s_ | -1414.8675 | 0.008039 | -1414.8595 | 0.114235 | 71.6824625 | 4.2921 | 1 | 2.3712 | 18.56 |
| t6(s11) | C_s_ | -1414.8621 | 0.007954 | -1414.8541 | 0.1196 | 75.049000 | 5.9642 | 0 | 2.0387 | 1.93 |
| t7(s14) | C_∞v_ | -1414.7903 | 0.010375 | -1414.7799 | 0.193751 | 121.578752 | 0.6005 | 2 | 2.3074 | 15.37 |

**Table S6** Point group, total energy (in a.u)**,** zero-point correction (in a.u), ZPVE corrected total energy (E+ZPVE; in a.u), relative energy with ZPVE corrections (ΔE + ZPVE; in a.u), relative energies with ZPVE corrections (ΔE + ZPVE) in kcal mol^-1^, absolute dipole moment (in Debye), and the number of imaginary frequencies (NImag) of FeC_2_O isomers in their corresponding singlet state computed at the TPSSh/6-311++G(2d,2p) theoretical level.

| **Isomer** | **Point group** | **Energy (a.u.)** | **ZPVE**  **(a.u.)** | **E+ZPVE**  **(a.u.)** | **∆E+ZPVE**  **(a.u)** | **∆E+ZPVE**  **(kcal/mol)** | **μ**  **(Debye)** | **Nimag** |
| --- | --- | --- | --- | --- | --- | --- | --- | --- |
| s1 | C*s* | -1415.0294 | 0.010602 | -1415.0188 | 0 | 0 | 3.7770 | 0 |
| s19 | C*1* | -1415.0133 | 0.010206 | -1415.0031 | 0.015714 | 9.860535 | 2.9282 | 0 |
| s4 | C*s* | -1414.9799 | 0.010779 | -1414.9691 | 0.049739 | 31.211223 | 3.9751 | 0 |
| s3 | C*∞v* | -1414.9753 | 0.011852 | -1414.9635 | 0.055359 | 34.737773 | 6.1208 | 1 |
| s6 | C*s* | -1414.9614 | 0.009337 | -1414.9521 | 0.066784 | 41.906960 | 3.1411 | 0 |
| s7 | C*s* | -1414.9602 | 0.008878 | -1414.9513 | 0.067500 | 42.356250 | 1.5053 | 1 |
| s8 | C*∞v* | -1414.9468 | 0.010018 | -1414.9368 | 0.082051 | 51.487003 | 4.2899 | 0 |
| s9 | C*1* | -1414.9361 | 0.009039 | -1414.9270 | 0.091795 | 57.601363 | 1.6087 | 0 |
| s17 | C*1* | -1414.9349 | 0.009404 | -1414.9255 | 0.093360 | 58.583400 | 1.1966 | 0 |
| s10 | C*2v* | -1414.9274 | 0.010315 | -1414.9171 | 0.101750 | 63.848125 | 0.0900 | 0 |
| s12 | C*s* | -1414.9115 | 0.008061 | -1414.9034 | 0.115399 | 72.412873 | 3.7179 | 0 |
| s11 | C*s* | -1414.9055 | 0.007537 | -1414.8979 | 0.120924 | 75.879810 | 5.5882 | 1 |
| s20 | C*s* | -1414.8942 | 0.007225 | -1414.8870 | 0.131877 | 82.752818 | 4.6101 | 1 |
| s15 | C*2v* | -1414.8915 | 0.007063 | -1414.8845 | 0.134372 | 84.318430 | 3.7372 | 2 |
| s14 | C*∞v* | -1414.8905 | 0.007923 | -1414.8826 | 0.136281 | 85.516328 | 2.6354 | 2 |
| s13 | C*s* | -1414.8872 | 0.009247 | -1414.8780 | 0.140880 | 88.402200 | 5.0228 | 0 |
| s5 | C*1* | -1414.8848 | 0.009717 | -1414.8751 | 0.143741 | 90.197478 | 3.3125 | 0 |
| s16 | C*∞v* | -1414.8795 | 0.010254 | -1414.8692 | 0.149634 | 93.895335 | 9.6363 | 0 |
| s18 | C*∞v* | -1414.8357 | 0.008328 | -1414.8274 | 0.191417 | 120.114170 | 3.3806 | 0 |

**Table S7** Point group, total energy (in a.u)**,** zero-point correction (in a.u), ZPVE corrected total energy (E+ZPVE; in a.u), relative energy with ZPVE corrections (ΔE + ZPVE; in a.u), relative energies with ZPVE corrections (ΔE + ZPVE) in kcal mol^-1^, absolute dipole moment (in Debye), and the number of imaginary frequencies (NImag) of FeC_2_O isomers in their corresponding singlet state computed at the TPSSh/SDD & 6-311++G(2d,2p) theoretical level.

| **Isomer** | **Point group** | **Energy (a.u.)** | **ZPVE**  **(a.u.)** | **E+ZPVE**  **(a.u.)** | **∆E+ZPVE**  **(a.u)** | **∆E+ZPVE**  **(kcal/mol)** | **μ (Debye)** | **Nimag** |
| --- | --- | --- | --- | --- | --- | --- | --- | --- |
| s1 | C*s* | -275.1683 | 0.010672 | -275.1576 | 0 | 0 | 3.6982 | 0 |
| s5 | C*1* | -275.1225 | 0.009405 | -275.1132 | 0.044486 | 27.914965 | 3.4550 | 0 |
| s3 | C*∞v* | -275.1208 | 0.012239 | -275.1086 | 0.049043 | 30.774483 | 4.5199 | 0 |
| s4 | C*s* | -275.1195 | 0.010722 | -275.1088 | 0.048822 | 30.635805 | 4.0122 | 0 |
| s6 | C*s* | -275.0989 | 0.009332 | -275.0896 | 0.068037 | 42.693218 | 3.0005 | 0 |
| s7 | C*s* | -275.0968 | 0.008830 | -275.0879 | 0.069665 | 43.714788 | 1.4926 | 1 |
| s8 | C*∞v* | -275.0846 | 0.010156 | -275.0745 | 0.083169 | 52.188548 | 4.2394 | 0 |
| s9 | C*1* | -275.0720 | 0.008925 | -275.0628 | 0.094788 | 59.479470 | 1.6068 | 0 |
| s17 | C*1* | -275.0706 | 0.009419 | -275.0612 | 0.096439 | 60.515473 | 1.1833 | 0 |
| s10 | C*2v* | -275.0619 | 0.009665 | -275.0522 | 0.105419 | 66.150423 | 0.3099 | 1 |
| s12 | C*s* | -275.0499 | 0.007960 | -275.0419 | 0.115672 | 72.584180 | 3.5864 | 0 |
| s11 | C*s* | -275.0459 | 0.007439 | -275.0385 | 0.119103 | 74.737133 | 5.5386 | 1 |
| s13 | C*s* | -275.0338 | 0.009331 | -275.0244 | 0.133192 | 83.577980 | 4.8717 | 0 |
| s15 | C*2v* | -275.0283 | 0.007052 | -275.0213 | 0.136346 | 85.557115 | 3.5056 | 2 |
| s14 | C*∞v* | -275.0235 | 0.007964 | -275.0156 | 0.142073 | 89.150808 | 2.6324 | 2 |
| s18 | C*∞v* | -274.9789 | 0.007548 | -274.9713 | 0.186296 | 116.900740 | 2.4770 | 0 |
| s16 | C*∞v* | -274.9744 | 0.011777 | -274.9626 | 0.194998 | 122.361250 | 9.6339 | 0 |

**Table S8** Point group, total energy (in a.u)**,** zero-point correction (in a.u), ZPVE corrected total energy (E+ZPVE; in a.u), relative energy with ZPVE corrections (ΔE + ZPVE; in a.u), relative energies with ZPVE corrections (ΔE + ZPVE) in kcal mol^-1^, absolute dipole moment (in Debye), and the number of imaginary frequencies (NImag) of FeC_2_O isomers in their corresponding singlet state computed at the ωB97XD/6-311++G(2d,2p) theoretical level.

| **Isomer** | **Point**  **group** | **Energy (a.u.)** | **ZPVE**  **(a.u.)** | **E+ZPVE**  **(a.u.)** | **∆E+ZPVE**  **(a.u)** | **∆E+ZPVE**  **(kcal/mol)** | **μ**  **(Debye)** | **Nimag** |
| --- | --- | --- | --- | --- | --- | --- | --- | --- |
| s1 | C*1* | -1414.9288 | 0.010845 | -1414.9179 | 0 | 0 | 2.9343 | 0 |
| s2 | C*s* | -1414.8997 | 0.011635 | -1414.888 | 0.02989 | 18.755975 | 6.891 | 1 |
| s3 | C*∞v* | -1414.8954 | 0.012250 | -1414.8831 | 0.034756 | 21.809390 | 6.6670 | 1 |
| s4 | C*s* | -1414.8919 | 0.011484 | -1414.8804 | 0.037533 | 23.551958 | 4.9506 | 0 |
| s5 | C*1* | -1414.8848 | 0.009717 | -1414.8751 | 0.042811 | 26.863903 | 2.451 | 0 |
| s6 | C*s* | -1414.8659 | 0.009513 | -1414.8564 | 0.061506 | 38.595015 | 3.3345 | 0 |
| s7 | C*s* | -1414.8632 | 0.009099 | -1414.8541 | 0.063847 | 40.063993 | 1.8826 | 1 |
| s8 | C*∞v* | -1414.8598 | 0.008967 | -1414.8508 | 0.067079 | 42.092073 | 5.2807 | 2 |
| s9 | C*1* | -1414.8266 | 0.009477 | -1414.8171 | 0.100828 | 63.26957 | 1.6914 | 0 |
| s10 | C*2v* | -1414.8219 | 0.010266 | -1414.8116 | 0.106288 | 66.69572 | 0.103 | 1 |
| s11 | C*s* | -1414.8086 | 0.007674 | -1414.801 | 0.116953 | 73.388008 | 5.8429 | 0 |
| s12 | C*s* | -1414.8054 | 0.007648 | -1414.7977 | 0.120165 | 75.403538 | 3.7151 | 0 |
| s13 | C*s* | -1414.8051 | 0.009826 | -1414.7953 | 0.1226 | 76.9315 | 4.7803 | 0 |
| s14 | C*∞v* | -1414.7817 | 0.008117 | -1414.7736 | 0.144299 | 90.547623 | 2.8304 | 4 |
| s15 | C*2v* | -1414.7776 | 0.00718 | -1414.7704 | 0.147537 | 92.579468 | 3.8127 | 2 |
| s16 | C*∞v* | -1414.7773 | 0.010135 | -1414.7672 | 0.150733 | 94.584958 | 11.6017 | 2 |

**Table S9** Point group, total energy (in a.u)**,** zero-point correction (in a.u), ZPVE corrected total energy (E+ZPVE; in a.u), relative energy with ZPVE corrections (ΔE + ZPVE; in a.u), relative energies with ZPVE corrections (ΔE + ZPVE) in kcal mol^-1^, absolute dipole moment (in Debye), and the number of imaginary frequencies (NImag) of FeC_2_O isomers in their corresponding singlet state computed at the ωB97XD/SDD & 6-311++G(2d,2p) theoretical level.

| **Isomer** | **Point**  **group** | **Energy  (a.u.)** | **ZPVE**  **(a.u.)** | **E+ZPVE**  **(a.u.)** | **∆E+ZPVE**  **(a.u)** | **∆E+ZPVE**  **(kcal/mol)** | **μ μ**  **(Debye)** | **Nimag** |
| --- | --- | --- | --- | --- | --- | --- | --- | --- |
| s1 | C*s* | -275.12473 | 0.010965 | -275.11377 | 0 | 0 | 3.6272 | 0 |
| s4 | C*s* | -275.09098 | 0.011369 | -275.07961 | 0.034155 | 21.432263 | 4.6249 | 0 |
| s5 | C*1* | -275.0782 | 0.009719 | -275.06848 | 0.045287 | 28.417593 | 2.392 | 0 |
| s6 | C*s* | -275.06041 | 0.009521 | -275.05089 | 0.062881 | 39.457828 | 3.2331 | 0 |
| s7 | C*s* | -275.05696 | 0.00911 | -275.04785 | 0.065922 | 41.366055 | 1.829 | 1 |
| s8 | C*∞v* | -275.05387 | 0.008984 | -275.04489 | 0.068882 | 43.223455 | 4.9913 | 2 |
| s3 | C*∞v* | -275.0436 | 0.012179 | -275.03143 | 0.082339 | 51.667723 | 6.7094 | 2 |
| s9 | C*1* | -275.01931 | 0.009699 | -275.00961 | 0.104159 | 65.359773 | 1.6342 | 0 |
| s17 | C*1* | -275.01913 | 0.009921 | -275.00921 | 0.104558 | 65.610145 | 1.2097 | 0 |
| s13 | C*s* | -275.01214 | 0.010016 | -275.00213 | 0.111641 | 70.054728 | 4.5705 | 0 |
| s11 | C*s* | -275.01062 | 0.008497 | -275.00212 | 0.11165 | 70.060375 | 5.8325 | 0 |
| s12 | C*s* | -275.00156 | 0.008339 | -274.99323 | 0.120543 | 75.640733 | 3.6396 | 0 |
| s10 | C*2v* | -275.00205 | 0.008971 | -274.99308 | 0.120691 | 75.733603 | 0.0032 | 1 |
| s15 | C*2v* | -274.97254 | 0.007416 | -274.96512 | 0.148645 | 93.274738 | 3.6066 | 2 |
| s14 | C*∞v* | -274.97195 | 0.008261 | -274.96368 | 0.150085 | 94.178338 | 2.8007 | 2 |
| s16 | C*∞v* | -274.97441 | 0.011777 | -274.96263 | 0.151135 | 94.837213 | 11.7509 | 0 |

**Table S10** ZPVE corrected relative energies of FeC_2_O isomers (in kcal mol^–1^) in their corresponding singlet electronic states obtained at different levels. The isomers are arranged in the order of energy computed at the ωB97XD/6–311++G(2d,2p) level of theory.

| **Isomers** | **ZPVE corrected relative energy in kcal mol^–1^** | | | | |
| --- | --- | --- | --- | --- | --- |
|  | **ωB97XD/**  **6–311++G(2d,2p)** | **TPSSh/**  **6–311++G(2d,2p)** | **TPSSh/SDD &**  **6– 311++G(2d,2p)** | **ωB97XD/SDD &**  **6–311++G(2d,2p)** |  |
| s1 | 0 | 0 | 0 | 0 |  |
| s2 | 18.76 | NA | NA | NA |  |
| s3 | 21.6 | 34.4 | 30.54 | 47.28 |  |
| s4 | 23.55 | 31.21 | 30.64 | 21.43 |  |
| s5 | 26.86 | 90.2 | 27.91 | 28.42 |  |
| s6 | 38.59 | 41.91 | 42.7 | 39.45 |  |
| s7 | 40.06 | 42.36 | 43.71 | 41.37 |  |
| s8 | 42.09 | 51.49 | 52.19 | 43.22 |  |
| s9 | 63.27 | 57.6 | 59.48 | 65.36 |  |
| s10 | 66.69 | 63.85 | 66.15 | 75.73 |  |
| s11 | 73.39 | 75.88 | 74.74 | 70.06 |  |
| s12 | 75.4 | 72.41 | 72.58 | 75.64 |  |
| s13 | 76.93 | 88.4 | 83.58 | 70.05 |  |
| s14 | 90.55 | 85.52 | 89.15 | 94.18 |  |
| s15 | 92.58 | 84.32 | 85.56 | 93.27 |  |
| s16 | 94.58 | 93.89 | 122.36 | 94.84 |  |
| s17 | NA | 58.58 | 60.52 | 65.61 |  |
| s18 | NA | 120.11 | 116.9 | NA |  |
| s19 | NA | 9.86 | NA | NA |  |
| s20 | NA | 82.75 | NA | NA |  |

^*^ NA (not applicable) indicates that the geometry did not converge at that level.

**Table S11** Optimized geometrical parameters (bond lengths in Å and bond angles in Degree) of FeC_2_O isomers in their corresponding quintet electronic state calculated at different levels of theory.

| **Isomers** | **Parameters** | **UTPSSh/**  **6-311++G(2d,2p)** | **UTPSSh/SDD&**  **6-311++G(2d,2p)** | **UωB97XD/**  **6- 311++G(2d,2p)** | **UωB97XD/SDD& 6-311++G(2d,2p)** |
| --- | --- | --- | --- | --- | --- |
| q1 | R(Fe,C2) | 1.749 | 1.746 | 1.753 | 1.747 |
|  | R(C2,C3) | 1.284 | 1.282 | 1.281 | 1.278 |
|  | R(C3,O) | 1.184 | 1.184 | 1.174 | 1.175 |
|  | θ(FeC2C3) | 180.000 | 180.000 | 180.000 | 180.000 |
|  | θ(C2C3O) | 180.000 | 180.000 | 180.000 | 180.000 |
| q2 | R(Fe,C2) | 2.039 | 2.077 | 2.103 | 2.104 |
|  | R(Fe,C3) | 2.039 | 2.077 | 2.103 | 2.094 |
|  | R(Fe,O) | 1.673 | 1.707 | 1.738 | 1.634 |
|  | R(C2,C3) | 1.275 | 1.270 | 1.260 | 1.307 |
|  | θ(FeC2C3) | 71.787 | 72.193 | 72.559 | 71.456 |
|  | θ(C2C3Fe) | 71.793 | 72.193 | 72.559 | 72.277 |
|  | θ(C3FeC2) | 36.420 | 35.614 | 34.883 | 36.267 |
|  | θ(C2FeO) | 161.774 | 162.193 | 162.559 | 162.131 |
|  | θ(C3FeO) | 161.764 | 162.193 | 162.559 | 161.543 |
| q3 | R(Fe,C2) | 1.906 | 1.916 | 1.919 | - |
|  | R(Fe,C3) | 1.906 | 1.916 | 1.919 | - |
|  | R(Fe,O) | 1.589 | 1.589 | 1.575 | - |
|  | R(C2,C3) | 1.289 | 1.289 | 1.277 | - |
|  | θ(FeC2C3) | 70.236 | 70.354 | 70.571 | - |
|  | θ(C2C3Fe) | 70.236 | 70.353 | 70.571 | - |
|  | θ(C3FeC2) | 39.529 | 39.291 | 38.858 | - |
|  | Φ(OFeC2C3) | 101.010 | 101.314 | 101.261 | - |
| q4 | R(C2,C3) | 1.229 | 1.302 | 1.219 | 1.218 |
|  | R(C3,Fe) | 1.953 | 1.935 | 1.974 | 1.969 |
|  | R(Fe,O) | 1.661 | 1.650 | 1.647 | 1.644 |
|  | θ(C2C3Fe) | 180.000 | 180.000 | 180.000 | 180.000 |
|  | θ(C3FeO) | 180.000 | 180.000 | 180.000 | 180.000 |
| q5 | R(Fe,C2) | 1.862 | 1.867 | 1.886 | 1.939 |
|  | R(Fe,C3) | 1.861 | 1.867 | 1.887 | 1.940 |
|  | R(C2,C3) | 1.431 | 1.428 | 1.406 | 1.407 |
|  | R(C2,O) | 1.421 | 1.421 | 1.405 | 1.399 |
|  | R(C3,O) | 1.423 | 1.423 | 1.405 | 1.399 |
|  | θ(FeC2C3) | 67.355 | 67.463 | 68.147 | 68.757 |
|  | θ(FeC3C2) | 67.441 | 67.569 | 68.072 | 68.697 |
|  | θ(C2FeC3) | 45.204 | 44.968 | 43.781 | 42.545 |
|  | θ(C2OC3) | 60.419 | 60.274 | 60.077 | 60.414 |
|  | θ(OC2C3) | 59.856 | 59.929 | 59.931 | 59.809 |
|  | θ(OC3C2) | 59.724 | 59.797 | 59.992 | 59.776 |
| q6 | R(Fe,C3) | 1.848 | 1.881 | 1.910 | 1.907 |
|  | R(C3,C2) | 1.375 | 1.373 | 1.366 | 1.363 |
|  | R(C2,O) | 1.479 | 1.498 | 1.446 | 1.457 |
|  | R(C3,O) | 1.414 | 1.393 | 1.397 | 1.386 |
|  | θ(C3C2O) | 59.282 | 57.821 | 59.500 | 58.748 |
|  | θ(C2OC3) | 56.678 | 56.579 | 57.403 | 57.219 |
|  | θ(OC3C2) | 64.040 | 65.600 | 63.097 | 64.033 |
|  | θ(OC3Fe) | 135.534 | 136.362 | 137.266 | 136.126 |
|  | θ(C2C3Fe) | 160.426 | 158.038 | 159.637 | 159.841 |
| q7 | R(Fe,O) | 1.740 | 1.867 | 1.731 | 1.821 |
|  | R(O,C2) | 1.257 | 1.242 | 1.254 | 1.208 |
|  | R(C2,C3) | 1.279 | 1.297 | 1.265 | 1.298 |
|  | θ(OC2C3) | 180.000 | 180.000 | 180.000 | 180.000 |
|  | θ(FeOC2) | 180.000 | 180.000 | 180.000 | 180.000 |
| q8 | R(Fe,C2) | - | - | 2.181 | 2.230 |
|  | R(Fe,C3) | - | - | 1.627 | 1.624 |
|  | R(Fe,O) | - | - | 2.238 | 2.334 |
|  | R(C2,O) | - | - | 1.156 | 1.150 |
|  | θ(FeC2O) | - | - | 77.601 | 80.472 |
|  | θ(C2OFe) | - | - | 72.098 | 70.461 |
|  | θ(OFeC2) | - | - | 30.302 | 29.0667 |
|  | θ(OFeC3) | - | - | 179.017 | 179.969 |
|  | θ(C2FeC3) | - | - | 150.681 | 150.964 |
| q9 | R(Fe,C2) | 1.970 | 1.987 | 2.023 | - |
|  | R(Fe,C3) | 1.969 | 1.987 | 2.023 | - |
|  | R(Fe,O) | 1.953 | 2.024 | 1.879 | - |
|  | R(C2,C3) | 1.410 | 1.423 | 1.358 | - |
|  | R(C2,O) | 1.552 | 1.521 | 1.608 | - |
|  | R(C3,O) | 1.552 | 1.521 | 1.608 | - |
|  | θ(C2FeO) | 46.627 | 44.536 | 48.512 | - |
|  | θ(FeC2O) | 66.124 | 69.054 | 61.032 | - |
|  | θ(FeOC2) | 67.249 | 66.449 | 70.455 | - |
|  | θ(OFeC3) | 46.627 | 44.536 | 48.512 | - |
|  | θ(FeOC3) | 67.249 | 66.449 | 70.455 | - |
|  | θ(FeC3O) | 66.124 | 69.015 | 61.032 | - |
|  | θ(C2FeC3) | 41.952 | 41.939 | 39.209 | - |
|  | θ(FeC2C3) | 69.024 | 69.030 | 70.396 | - |
|  | θ(FeC3C2) | 69.024 | 69.030 | 70.395 | - |
| q10 | R(C2,Fe) | 1.684 | 1.722 | 1.741 | 1.813 |
|  | R(Fe,C3) | 1.971 | 1.962 | 1.890 | 1.869 |
|  | R(C3,O) | 1.141 | 1.140 | 1.135 | 1.135 |
|  | θ(C2FeC3) | 180.000 | 180.000 | 180.000 | 180.000 |
|  | θ(FeC3O) | 180.000 | 180.000 | 180.000 | 180.000 |
| q11 | R(Fe,C2) | 1.773 | 1.793 | - | 1.786 |
|  | R(C2,C3 | 1.300 | 1.312 | - | 1.303 |
|  | R(C3,O) | 1.181 | 1.180 | - | 1.172 |
|  | θ(FeC2C3) | 134.542 | 115.728 | - | 120.753 |
|  | θ(C2C3O) | 174.368 | 172.033 | - | 173.637 |
| q12 | R(C2,Fe) | 1.697 | 1.740 | - | - |
|  | R(Fe,C3) | 1.882 | 1.880 | - | - |
|  | R(C3,O) | 1.144 | 1.144 | - | - |
|  | θ(C2FeC3) | 127.574 | 130.045 | - | - |
|  | θ(FeC3O) | 178.736 | 178.445 | - | - |
| q13 | R(Fe,C2) | 1.715 | 1.713 | - | - |
|  | R(C2,O) | 1.273 | 1.273 | - | - |
|  | R(O,C3) | 1.238 | 1.236 | - | - |
|  | θ(FeC2O) | 180.000 | 180.000 | - | - |
|  | θ(C2OC3) | 180.000 | 180.000 | - | - |
| q14 | R(Fe,C2) | - | 2.017 | - | - |
|  | R(Fe,C3) | - | 2.017 | - | - |
|  | R(C2,O) | - | 1.363 | - | - |
|  | R(C3,O) | - | 1.363 | - | - |
|  | θ(C2FeC3) | - | 66.495 | - | - |
|  | θ(C2OC3) | - | 108.480 | - | - |
|  | θ(FeC3O) | - | 92.512 | - | - |
|  | θ(OC2Fe) | - | 92.512 | - | - |

**Table S12** Optimized geometrical parameters (bond lengths in Å and bond angles in Degree) of FeC_2_O isomers in their corresponding singlet electronic state calculated at different levels.

| **Isomers** | **Parameters** | **TPSSh/**  **6- 311++G(2d,2p)** | **TPSSh/SDD&**  **6-311++G(2d,2p)** | **ωB97XD/**  **6- 311++G(2d,2p)** | **ωB97XD/SDD&**  **6-311++G(2d,2p)** |
| --- | --- | --- | --- | --- | --- |
| s1 | R(C2,Fe) | 1.519 | 1.518 | 1.493 | 1.494 |
|  | R(Fe,C3) | 1.764 | 1.753 | 1.779 | 1.763 |
|  | R(C3,O) | 1.153 | 1.153 | 1.141 | 1.141 |
|  | θ(C2FeC3) | 96.393 | 95.226 | 97.393 | 95.639 |
|  | θ(FeC3O) | 171.911 | 173.538 | 171.3 | 172.953 |
| s2 | R(Fe,C2) | - | - | 1.701 | - |
|  | R(C2,C3) | - | - | 1.328 | - |
|  | R(C3,O) | - | - | 1.171 | - |
|  | R(Fe,C3) | - | - | 2.288 | - |
|  | θ(FeC2C3) | - | - | 35.126 | - |
|  | θ(C2C3Fe) | - | - | 47.505 | - |
|  | θ(C2FeC3) | - | - | 35.126 | - |
|  | θ(FeC3O) | - | - | 143.284 | - |
| s3 | R(Fe,C2) | 1.723 | 1.686 | 1.724 | 1.69 |
|  | R(C2,C3) | 1.292 | 1.309 | 1.291 | 1.296 |
|  | R(C3,O) | 1.181 | 1.167 | 1.17 | 1.162 |
|  | θ(FeC2C3) | 179.886 | 179.932 | 179.7 | 179.705 |
|  | θ(C2C3O) | 179.928 | 179.839 | 179.949 | 179.924 |
| s4 | R(Fe,C2) | 1.816 | 1.831 | 1.867 | 1.884 |
|  | R(Fe,C3) | 1.782 | 1.782 | 1.803 | 1.812 |
|  | R(Fe,O) | 1.935 | 1.958 | 1.99 | 2.008 |
|  | R(C2,C3) | 1.36 | 1.352 | 1.318 | 1.312 |
|  | R(C3,O) | 1.25 | 1.249 | 1.237 | 1.239 |
|  | θ(FeC2C3) | 66.456 | 66.124 | 66.385 | 66.315 |
|  | θ(C2FeC3) | 44.391 | 43.931 | 42.047 | 41.535 |
|  | θ(FeC3C2) | 69.152 | 69.945 | 71.567 | 72.149 |
|  | θ(FeC3O) | 77.132 | 78.321 | 79.351 | 79.884 |
|  | θ(C3FeO) | 39.039 | 38.643 | 37.675 | 37.418 |
|  | θ(FeOC3) | 63.829 | 63.036 | 62.973 | 62.697 |
| s5 | R(Fe,C2) | 1.958 | 1.942 | 1.958 | 1.96 |
|  | R(Fe,C3) | 1.482 | 1.504 | 1.482 | 1.479 |
|  | R(Fe,O) | 2.012 | 1.985 | 2.012 | 2.013 |
|  | R(C2,O) | 1.178 | 1.197 | 1.178 | 1.177 |
|  | θ(FeC2O) | 75.273 | 74.225 | 75.273 | 75.249 |
|  | θ(C2OFe) | 70.248 | 70.307 | 70.248 | 70.314 |
|  | θ(OFeC2) | 34.479 | 35.468 | 34.479 | 34.437 |
|  | Φ(C3FeOC2) | 84.991 | 85.052 | 84.991 | 85.344 |
| s6 | R(C2, Fe) | 1.504 | 1.502 | 1.48 | 1.479 |
|  | R(Fe,O) | 1.866 | 1.87 | 1.953 | 1.954 |
|  | R(O,C3) | 1.153 | 1.151 | 1.134 | 1.133 |
|  | θ(C2FeO) | 110.444 | 109.157 | 111.856 | 110.399 |
|  | θ(FeOC3) | 168.95 | 172.062 | 168.67 | 171.643 |
| s7 | R(Fe,C2) | 2.287 | 2.302 | 2.353 | 2.371 |
|  | R(Fe,C3) | 1.498 | 1.493 | 1.475 | 1.471 |
|  | R(Fe,O) | 1.912 | 1.92 | 1.979 | 1.988 |
|  | R(C2,O) | 1.178 | 1.176 | 1.154 | 1.152 |
|  | θ(FeC2O) | 56.638 | 56.428 | 57.047 | 56.758 |
|  | θ(C2OFe) | 92.405 | 92.908 | 93.653 | 94.244 |
|  | θ(OFeC2) | 30.957 | 30.668 | 29.3 | 28.998 |
|  | θ(OFeC3) | 114.742 | 114.006 | 114.139 | 113.431 |
|  | θ(C2FeC3) | 145.7 | 144.674 | 143.439 | 142.429 |
| s8 | R(C2,Fe) | 1.52 | 1.514 | 1.485 | 1.481 |
|  | R(Fe,C3) | 2.139 | 2.145 | 2.237 | 2.234 |
|  | R(C3,O) | 1.13 | 1.13 | 1.119 | 1.119 |
|  | θ(C2FeC3) | 180 | 180 | 180 | 180 |
|  | θ(FeC3O) | 180 | 180 | 180 | 180 |
| s9 | R(Fe,C2) | 1.824 | 1.822 | 1.835 | 1.83 |
|  | R(Fe,C3) | 1.824 | 1.823 | 1.835 | 1.83 |
|  | R(Fe,O) | 1.547 | 1.543 | 1.522 | 1.517 |
|  | R(C2,C3) | 1.305 | 1.305 | 1.283 | 1.285 |
|  | θ(FeC2C3) | 69.036 | 69.022 | 69.529 | 69.453 |
|  | θ(C2C3Fe) | 69.044 | 69.004 | 69.527 | 69.449 |
|  | θ(C3FeC2) | 41.919 | 41.974 | 40.944 | 41.099 |
|  | Φ(OFeC2C3) | 111.851 | 111.121 | 114.765 | 113.544 |
| s10 | R(Fe,C2) | 1.853 | 1.852 | 1.854 | 1.877 |
|  | R(Fe,C3) | 1.853 | 1.852 | 1.854 | 1.877 |
|  | R(Fe,O) | 1.537 | 1.533 | 1.514 | 1.613 |
|  | R(C2,C3) | 1.275 | 1.275 | 1.265 | 1.303 |
|  | θ(FeC2C3) | 69.88 | 69.87 | 70.048 | 69.693 |
|  | θ(C2C3Fe) | 69.88 | 69.87 | 70.048 | 69.693 |
|  | θ(C3FeC2) | 40.24 | 40.26 | 39.904 | 40.615 |
|  | θ(C2FeO) | 159.88 | 159.87 | 160.048 | 159.693 |
|  | θ(C3FeO) | 159.88 | 159.87 | 159.693 | 159.693 |
| s11 | R(Fe,C2) | 1.823 | 1.833 | 1.887 | 1.912 |
|  | R(Fe,C3) | 1.823 | 1.833 | 1.887 | 1.912 |
|  | R(Fe,O) | 1.874 | 1.874 | 1.862 | 1.857 |
|  | R(C2,C3) | 1.463 | 1.454 | 1.4 | 1.39 |
|  | R(C2,O) | 1.517 | 1.52 | 1.51 | 1.519 |
|  | R(C3,O) | 1.517 | 1.52 | 1.51 | 1.519 |
|  | θ(C2FeO) | 48.431 | 48.398 | 47.477 | 47.495 |
|  | θ(FeC2O) | 67.553 | 67.202 | 65.375 | 64.36 |
|  | θ(FeOC2) | 64.016 | 64.4 | 67.148 | 68.144 |
|  | θ(OFeC3) | 48.431 | 48.398 | 47.477 | 47.495 |
|  | θ(FeOC3) | 64.016 | 64.4 | 67.148 | 68.144 |
|  | θ(FeC3O) | 67.553 | 67.202 | 65.375 | 64.36 |
|  | θ(C2FeC3) | 47.336 | 46.721 | 43.537 | 42.617 |
|  | θ(FeC2C3) | 66.332 | 66.64 | 68.232 | 68.692 |
|  | θ(FeC3C2) | 66.332 | 66.64 | 68.232 | 68.692 |
| s12 | R(Fe,C2) | 1.667 | 1.664 | 1.679 | 1.677 |
|  | R(Fe,C3) | 1.665 | 1.665 | 1.679 | 1.677 |
|  | R(C2,C3) | 1.796 | 1.794 | 1.657 | 1.669 |
|  | R(C2,O) | 1.405 | 1.401 | 1.385 | 1.381 |
|  | R(C3,O) | 1.402 | 1.404 | 1.386 | 1.383 |
|  | θ(FeC2C3) | 57.33 | 57.42 | 60.435 | 60.177 |
|  | θ(FeC3C2) | 57.427 | 57.341 | 60.423 | 60.146 |
|  | θ(C2FeC3) | 65.243 | 65.238 | 59.142 | 59.677 |
|  | θ(C2OC3) | 79.6 | 79.552 | 73.46 | 74.297 |
|  | θ(OC2C3) | 50.128 | 50.293 | 53.293 | 52.884 |
|  | θ(OC3C2) | 50.271 | 50.155 | 53.247 | 52.819 |
| s13 | R(Fe,C3) | 1.714 | 1.705 | 1.714 | 1.702 |
|  | R(C3,C2) | 1.447 | 1.446 | 1.454 | 1.454 |
|  | R(C2,O) | 1.342 | 1.342 | 1.319 | 1.32 |
|  | R(C3,O) | 1.457 | 1.456 | 1.425 | 1.42 |
|  | θ(C3C2O) | 62.852 | 62.829 | 61.61 | 61.37 |
|  | θ(C2OC3) | 62.104 | 62.105 | 63.882 | 63.968 |
|  | θ(OC3C2) | 55.043 | 55.066 | 54.508 | 54.662 |
|  | θ(OC3Fe) | 138.769 | 137.724 | 140.847 | 139.892 |
|  | θ(C2C3Fe) | 166.188 | 167.21 | 164.645 | 165.447 |
| s14 | R(C2,C3) | 1.302 | 1.302 | 1.294 | 1.294 |
|  | R(C3,Fe) | 1.783 | 1.78 | 1.769 | 1.765 |
|  | R(Fe,O) | 1.546 | 1.544 | 1.521 | 1.517 |
|  | θ(C2C3Fe) | 180 | 180 | 180 | 180 |
|  | θ(C3FeO) | 180 | 180 | 180 | 180 |
| s15 | R(Fe,C2) | 1.684 | 1.683 | 1.677 | 1.674 |
|  | R(Fe,C3) | 1.684 | 1.683 | 1.677 | 1.674 |
|  | R(Fe,O) | 1.905 | 1.899 | 1.886 | 1.881 |
|  | R(C2,O) | 1.451 | 1.452 | 1.425 | 1.426 |
|  | R(C3,O) | 1.451 | 1.452 | 1.425 | 1.426 |
|  | θ(FeC2O) | 74.42 | 74.175 | 74.416 | 74.234 |
|  | θ(FeOC2) | 58.38 | 58.495 | 58.898 | 58.909 |
|  | θ(C2FeO) | 47.2 | 47.33 | 46.685 | 46.857 |
|  | θ(FeC3O) | 74.42 | 74.175 | 74.416 | 74.234 |
|  | θ(FeOC3) | 58.38 | 58.495 | 58.898 | 58.909 |
|  | θ(C3FeO) | 47.2 | 47.33 | 46.685 | 46.857 |
| s16 | R(Fe,O) | 1.65 | 1.643 | 1.631 | 1.623 |
|  | R(O,C2) | 1.255 | 1.254 | 1.263 | 1.264 |
|  | R(C2,C3) | 1.277 | 1.277 | 1.258 | 1.257 |
|  | θ(OC2C3) | 180 | 180 | 180 | 180 |
|  | θ(FeOC2) | 180 | 180 | 180 | 180 |
| s17 | R(Fe,C2) | 1.935 | 1.964 | - | 1.939 |
|  | R(Fe,C3) | 1.761 | 1.745 | - | 1.758 |
|  | R(Fe,O) | 1.536 | 1.531 | - | 1.508 |
|  | R(C2,C3) | 1.293 | 1.292 | - | 1.277 |
|  | θ(FeC2C3) | 62.44 | 60.704 | - | 62.335 |
|  | θ(C2C3Fe) | 76.964 | 79.048 | - | 77.628 |
|  | θ(C3FeC2) | 40.596 | 40.248 | - | 40.036 |
|  | θ(C2FeO) | 169.665 | 168.008 | - | 169.832 |
|  | θ(C3FeO) | 129.077 | 127.765 | - | 129.803 |
| s18 | R(Fe,C2) | 1.617 | 1.592 | - | - |
|  | R(C2,O) | 1.355 | 1.416 | - | - |
|  | R(O,C3) | 1.19 | 1.171 | - | - |
|  | θ(FeC2O) | 180 | 180 | - | - |
|  | θ(C2OC3) | 180 | 180 | - | - |
| s19 | R(C2,Fe) | 1.513 | - | - | - |
|  | R(Fe,C3) | 1.857 | - | - | - |
|  | R(C3,O) | 1.155 | - | - | - |
|  | θ(C2FeC3) | 123.553 | - | - | - |
|  | θ(FeC3O) | 169.282 | - | - | - |
| s20 | R(Fe,C2) | 1.801 | - | - | - |
|  | R(Fe,C3) | 1.805 | - | - | - |
|  | R(C2,C3) | 1.48 | - | - | - |
|  | R(C2,O) | 1.384 | - | - | - |
|  | R(C3,O) | 1.382 | - | - | - |
|  | θ(FeC2C3) | 65.914 | - | - | - |
|  | θ(FeC3C2) | 65.62 | - | - | - |
|  | θ(C2FeC3) | 48.465 | - | - | - |
|  | θ(C2OC3) | 64.708 | - | - | - |
|  | θ(OC2C3) | 57.58 | - | - | - |
|  | θ(OC3C2) | 57.712 | - | - | - |

**Table S13** Relative energies (ΔE + ZPVE) of FeC_2_O isomers in their quintet electronic state optimized at UωB97XD functional with 6-311++G(2d,2p) basis sets, and single-point energy calculation at the CCSD(T) with 6-311++G(2d,2p) basis sets, along with the T_1_ diagnostic values.

| **Isomer** | **Relative Energies** | | **T_1_ diagnostic value** |
| --- | --- | --- | --- |
|  | **UωB97XD/6-311++g(2d,2p)** | **CCSD(T)/6-311++g(2d,2p) //** |  |
|  |  | **UωB97XD/6-311++g(2d,2p)** |  |
| q1 | 0 | 0 | 0.0584 |
| q2 | 66.7 | 67.58 | 0.0583 |
| q3 | 67.87 | 53.03 | 0.1179 |
| q4 | 72.24 | 50.2 | 0.0714 |
| q5 | 74.29 | 75.51 | 0.0606 |
| q6 | 74.58 | 77.69 | 0.0360 |
| q7 | 75.98 | 60.74 | 0.5698 |
| q8 | 77.33 | 61.76 | 0.1246 |
| q9 | 78.71 | 76.31 | 0.1481 |
| q10 | 85.48 | 47.77 | 0.0892 |

**Table S14** Comparison of the relative energies (ΔE + ZPVE) of FeC_2_O isomers in their triplet electronic state optimized at UωB97XD functional with 6-311++G(2d,2p) basis sets, and single-point energy calculation at the CCSD(T) with 6-311++G(2d,2p) basis sets, along with the T_1_ diagnostic values.

| **Isomer** | **Relative Energies** | | **T_1_ diagnostic value** |
| --- | --- | --- | --- |
|  | **UωB97XD/**  **6-311++g(2d,2p)** | **CCSD(T)/6-311++g(2d,2p) //** |  |
|  |  | **UωB97XD/6-311++g(2d,2p)** |  |
| t1 | 0 | 45.75 | 0.0795 |
| t2 | 32.26 | 0 | 0.0895 |
| t3 | 34.95 | 22.37 | 0.0496 |
| t4 | 48.72 | 65.21 | 0.0267 |
| t5 | 71.68 | 78.85 | 0.1148 |
| t6 | 75.05 | 62.88 | 0.0529 |
| t7 | 121.58 | 90.72 | 0.0817 |

**Table S15** Comparison of the relative energies (ΔE + ZPVE) of FeC_2_O isomers in their singlet electronic state optimized at ωB97XD functional with 6-311++G(2d,2p) basis sets, and single-point energy calculation at the CCSD(T) with 6-311++G(2d,2p) basis sets, along with the T_1_ diagnostic values.

| **Isomer** | **Relative Energies** | | **T_1_ diagnostic value** |
| --- | --- | --- | --- |
|  | **ωB97XD/6-311++g(2d,2p)** | **CCSD(T)/6-311++g(2d,2p) //** |  |
|  |  | **ωB97XD/6-311++g(2d,2p)** |  |
| s1 | 0 | 0 | 0.1303 |
| s2 | 18.76 | 33.62 | 0.1281 |
| s3 | 21.81 | 33.14 | 0.1046 |
| s4 | 23.55 | 36.59 | 0.1084 |
| s5 | 26.86 | 25.35 | 0.1293 |
| s6 | 38.59 | 18.22 | 0.2204 |
| s7 | 40.06 | NA | NA |
| s8 | 42.09 | 45.35 | 0.1045 |
| s9 | 63.27 | 58.81 | 0.1368 |
| s10 | 66.69 | 63.28 | 0.0845 |
| s11 | 73.39 | 89.51 | 0.0902 |
| s12 | 75.40 | NA | NA |
| s13 | 76.93 | 109.22 | 0.1094 |
| s14 | 90.55 | 91.22 | 0.0587 |
| s15 | 92.58 | 81.51 | 0.1661 |
| s16 | 94.58 | 76.58 | 0.1255 |

^*^ NA (not applicable) indicates that the geometry did not converge at that level.

**Table S16** Comparison of the relative energies (ΔE + ZPVE) of FeC_2_O isomers in their quintet electronic state optimized at UωB97XD/6-311++G(2d,2p), and CCSD/6-311++G(2d,2p) levels of theory.

| **Isomers** | **UωB97XD/6-311++g(2d,2p)** | **CCSD/6-311++g(2d,2p)** |
| --- | --- | --- |
| q1 | 0 | 0 |
| q2 | 66.7 | 62.08 |
| q4 | 72.24 | 49.56 |
| q5 | 74.29 | 76.19 |
| q14 | - | 107.99 |
| q13 | - | 113.93 |

**Table S17** Comparison of the relative energies (ΔE + ZPVE) of FeC_2_O isomers in their quintet electronic state optimized at UωB97XD/6-311++G(2d,2p), and CASSCF/def2-tzvp levels of theory.

| **Isomer** | **Relative Energies** | |
| --- | --- | --- |
|  | **(U)ωB97XD/6-311++g(2d,2p)** | **CASSCF (final energy)** |
|  |  |  |
| q1 | 0 | 0 |
| q2 | 66.7 | 70.91 |
| q3 | 67.87 | 131.3 |
| q4 | 72.24 | 107.04 |
| q5 | 74.29 | 85.59 |
| q6 | 74.58 | 73.33 |
| q7 | 75.98 | 28.87 |
| q8 | 77.33 | 76.11 |
| q9 | 78.71 | 116.49 |
| q10 | 85.48 | 61.37 |

**Table S18** Spin-resolved atomic composition (%) of the frontier molecular orbitals of q1 at the UωB97XD/6–311++G(2d,2p) level.

| Orbital | Fe (%) | C1 (%) | C2 (%) | O (%) |
| --- | --- | --- | --- | --- |
| α-HOMO | 6.77 | 12.55 | 55.76 | 24.92 |
| α-LUMO | 86.32 | 1.45 | 11.98 | 0 |
| β-HOMO | 31.01 | 2.57 | 49.39 | 17.02 |
| β-LUMO | 94.08 | 1.122 | 4.55 | 0 |

**Table S19** The Cartesian coordinates (in Angstrom units) of isomers of FeC_2_O in their quintet electronic state obtained at different levels of theory.

| q1; UTPSSh/6-311++G(2d,2p) | q1; UTPSSh/SDD & 6-311++G(2d,2p) |
| --- | --- |
| C 0.000000 0.000000 1.676528  C 0.000000 0.000000 0.391594  Fe 0.000000 0.000000 -1.357297  O 0.000000 0.000000 2.860125 | C 0.000000 0.000000 1.672488  C 0.000000 0.000000 0.390504  Fe 0.000000 0.000000 -1.355080  O 0.000000 0.000000 2.856765 |
| q1; UωB97XD /6-311++G(2d,2p) | q1; UωB97XD /SDD & 6311++G(2d,2p) |
| C 0.000000 0.000000 1.678552  C 0.000000 0.000000 0.397095  Fe 0.000000 0.000000 -1.356870  O 0.000000 0.000000 2.853091 | C 1.516793 1.273023 -0.001214  C 1.516793 1.273023 1.277132  Fe 1.516793 1.273023 3.023756  O 1.516793 1.273023 -1.176415 |
| q2; UTPSSh/6-311++G(2d,2p) | q2; UTPSSh/SDD & 6-311++G(2d,2p) |
| Fe -2.221287 -0.665290 0.006550  O -3.891900 -0.576978 -0.010139  C -0.320651 -1.404431 0.003083  C -0.253034 -0.131734 -0.012392 | Fe -2.221018 0.731311 0.075757  C -2.221018 1.366459 -1.901645  C -2.221018 0.096163 -1.901645  O -2.221018 0.731311 1.782447 |
| q2; UωB97XD/6-311++G(2d,2p) | q2; UωB97XD/SDD & 6-311++G(2d,2p) |
| Fe -0.271189 0.726642 0.005963  C 0.495827 -1.043284 0.005963  C -0.954094 -1.077407 0.005963  O -0.308871 2.327799 0.005963 | Fe -1.025191 0.423481 -0.008356  O -2.657576 0.338210 0.010923  C 0.924448 1.188805 -0.005291  C 1.008900 -0.115152 0.014499 |
| q3; UTPSSh/6-311++G(2d,2p) | q3; UTPSSh/SDD & 6-311++G(2d,2p) |
| C -1.079712 -0.100314 0.048643  O 1.841594 -0.746039 0.269156  C -1.079545 -1.389403 0.039773  Fe 0.563794 -0.739699 -0.674810 | C -1.538057 0.284687 0.044865  O -4.481401 0.928842 0.271314  C -1.538096 1.573371 0.045295  Fe -3.197680 0.929202 -0.664520 |
| q3; UωB97XD/6-311++G(2d,2p) | q4; UTPSSh/6-311++G(2d,2p) |
| C -0.950182 0.235272 0.047046  O 1.989248 -0.404452 0.262591  C -0.950009 -1.041877 0.038258  Fe 0.716271 -0.398204 -0.665133 | C -1.436512 -0.330444 -0.053976  C -1.436512 -0.330444 -1.283111  Fe -1.436512 -0.330444 -3.236283  O -1.436512 -0.330444 -4.897474 |
| q4; UTPSSh/SDD & 6-311++G(2d,2p) | q4; UωB97XD/6-311++G(2d,2p) |
| C -1.744312 0.460455 -0.106055  C -1.744312 0.460455 1.196481  Fe -1.744312 0.460455 3.131152  O -1.744312 0.460455 4.788734 | C -0.227766 1.832972 0.058231  C -0.227766 1.832972 -1.160544  Fe -0.227766 1.832972 -3.134359  O -0.227766 1.832972 -4.781947 |
| q4; UωB97XD/ SDD&6-311++G(2d,2p) | q5; UTPSSh/6-311++G(2d,2p) |
| C 0.000000 0.000000 -2.770639  C 0.000000 0.000000 -1.552583  Fe 0.000000 0.000000 0.416845  O 0.000000 0.000000 2.061351 | Fe 0.464699 0.853528 0.000000  C 1.180978 -0.865166 0.000000  C -0.249789 -0.864651 0.000000  O 0.467056 -2.093567 0.000000 |
| q5; UTPSSh/SDD & 6-311++G(2d,2p) | q5; UωB97XD/6-311++G(2d,2p) |
| C -1.516286 0.981451 0.000000  C -2.944174 0.980333 0.000000  Fe -2.229694 2.705888 0.000000  O -2.231159 -0.248905 0.000000 | C -1.631453 2.583941 0.000000  C -3.038103 2.582093 0.000000  Fe -2.333815 0.832595 0.000000  O -2.335505 3.799332 0.000000 |
| q5; UωB97XD/SDD& 6-311++G(2d,2p) | q6; UTPSSh/6-311++G(2d,2p) |
| C -0.506661 0.059724 0.000000  C -1.914401 0.060372 0.000000  Fe -1.210796 1.867940 0.000000  O -1.211559 -1.148975 0.000000 | Fe 0.291140 -0.918819 0.000000  C -0.109279 0.885648 0.000000  C 0.059820 2.250428 0.000000  O -1.295584 1.656710 0.000000 |
| q6; UTPSSh/ SDD & 6-311++G(2d,2p) | q6;U ωB97XD/6-311++G(2d,2p) |
| Fe -1.439743 0.458525 0.000000  C -0.966788 2.279652 0.000000  C -1.143750 3.641463 0.000000  O 0.216710 3.013553 0.000000 | Fe -1.637067 -0.587770 0.000000  C -1.236158 1.279494 0.000000  C -1.432091 2.631569 0.000000  O -0.093688 2.083890 0.000000 |
| q6; UωB97XD/SDD& 6-311++G(2d,2p) | q7; UTPSSh/6-311++G(2d,2p) |
| Fe -0.349418 -1.165371 0.000000  C 0.032826 0.702364 0.000000  C -0.170831 2.050215 0.000000  O 1.174310 1.488638 0.000000 | C -1.863489 -0.157083 -0.022689  O -1.863489 -0.157083 2.512941  C -1.863489 -0.157083 1.256299  Fe -1.863489 -0.157083 4.252935 |
| q7; UTPSSh/SDD & 6-311++G(2d,2p) | q7; UωB97XD/6-311++G(2d,2p) |
| C -1.105092 0.352113 -0.065115  O -1.105092 0.352113 2.474849  C -1.105092 0.352113 1.231912  Fe -1.105092 0.352113 4.341820 | C -1.594360 2.917570 -0.010861  O -1.594360 2.917570 2.508662  C -1.594360 2.917570 1.254145  Fe -1.594360 2.917570 4.239315 |
| q7; UωB97XD/SDD &6-311++G(2d,2p) | q8; UωB97XD/6-311++G(2d,2p) |
| C -1.885157 0.048754 -0.052363  O -1.885157 0.048754 2.453594  C -1.885157 0.048754 1.246022  Fe -1.885157 0.048754 4.274770 | Fe -1.212209 1.874617 0.000000  C -2.999933 0.625514 0.000000  C -0.505494 3.340520 0.000000  O -2.149534 -0.158063 0.000000 |
| q8; UωB97XD/SDD& 6-311++G(2d,2p) | q9; UTPSSh/6-311++G(2d,2p) |
| Fe -0.198737 -1.225138 0.000000  C -1.985414 -2.560356 0.000000  C 0.466834 0.256350 0.000000  O -1.154127 -3.354769 0.000000 | O -1.636088 1.090775 0.000000  C -2.964383 0.705201 0.705055  C -2.964383 0.705201 -0.705055  Fe -2.940676 2.544102 0.000000 |
| q9; UTPSSh/SDD & 6-311++G(2d,2p) | q9; UωB97XD/6-311++G(2d,2p) |
| O -1.396171 0.453524 0.000000  C -2.698370 0.120695 0.711285  C -2.698370 0.120695 -0.711285  Fe -2.729965 1.976344 0.000000 | O -1.955663 2.399690 0.000000  C -3.321964 1.890253 0.678932  C -3.321964 1.890253 -0.678932  Fe -3.215230 3.793473 0.000000 |
| q10; UTPSSh/6-311++G(2d,2p) | q10; UTPSSh/SDD & 6-311++G(2d,2p) |
| O -0.888331 -0.189599 0.022872  C -0.888331 -0.189599 1.164071  C -0.888331 -0.189599 4.818587  Fe -0.888331 -0.189599 3.134903 | O 0.000000 0.000000 -2.591265  C 0.000000 0.000000 -1.450965  C 0.000000 0.000000 2.233133  Fe 0.000000 0.000000 0.510686 |
| q10; UωB97XD /6-311++G(2d,2p) | q10; UωB97XD/SDD& 6311++G(2d,2p) |
| O -2.570499 1.225597 -0.054547  C -2.570499 1.225597 1.080503  C -2.570499 1.225597 4.711364  Fe -2.570499 1.225597 2.970219 | O -0.541712 0.622969 0.087512  C -0.541712 0.622969 1.222348  C -0.541712 0.622969 4.904750  Fe -0.541712 0.622969 3.091548 |
| q11; UTPSSh/6-311++G(2d,2p) | q11; UTPSSh/SDD & 6-311++G(2d,2p) |
| O 0.691193 1.777884 -0.009856  C 1.534929 0.952107 -0.009856  C 2.548960 0.138218 -0.009856  Fe 2.727901 -1.626276 -0.009856 | O -1.509785 -0.184964 0.000000  C -0.592101 0.557412 0.000000  C 0.532671 1.233359 0.000000  Fe 0.367821 3.018589 0.000000 |
| q11; UωB97XD/SDD&6311++G(2d,2p) | q12; UTPSSh/6-311++G(2d,2p) |
| O -1.107047 -1.821525 0.000000  C -0.185894 -1.097801 0.000000  C 0.937245 -0.427897 0.000000  Fe 0.723918 1.341722 0.000000 | C -1.334949 -0.370489 -0.000747  C 1.760170 0.490019 -0.001265  Fe 0.523262 -0.671952 -0.000418  O -2.459890 -0.162414 -0.000941 |
| q12; UTPSSh/SDD & 6-311++G(2d,2p) | q13; UTPSSh/6-311++G(2d,2p) |
| Fe -0.630090 0.948815 0.000000  C -1.055568 -0.882581 0.000000  C 0.920412 1.737678 0.000000  O -1.284049 -2.003211 0.000000 | C -0.399868 -0.000010 0.002138  Fe 1.315533 0.000034 -0.007035  O -1.673085 -0.000043 0.008947  C -2.910822 -0.000075 0.015566 |
| q13; UTPSSh/SDD & 6-311++G(2d,2p) | q14; UTPSSh/SDD & 6-311++G(2d,2p) |
| C -2.036836 0.752979 0.085701  Fe -2.036836 0.752979 -1.627300  O -2.036836 0.752979 1.358665  C -2.036836 0.752979 2.594537 | O -1.278440 0.406284 -0.162318  Fe -1.278440 0.406284 2.320769  C -1.278440 1.512064 0.634015  C -1.278440 -0.699497 0.634015 |

**Table S20** The Cartesian coordinates (in Angstrom units) of isomers of FeC_2_O in their triplet electronic state obtained at the UωB97XD/6-311++G(2d,2p) theoretical level.

| t1 | t2 |
| --- | --- |
| O -2.331887 0.401302 0.000000  Fe -1.619440 3.211295 0.000000  C -0.154194 2.917005 0.000000  C -2.134756 1.525265 0.000000 | O -3.241990 1.768578 -0.061745  C -2.009697 1.637375 -0.061745  C -0.792066 2.126586 -0.061745  Fe -2.137325 3.445177 -0.061745 |
| t3 | t4 |
| C -1.594360 2.917570 0.000000  O -1.594360 2.917570 2.531972  C -1.594360 2.917570 1.277167  Fe -1.594360 2.917570 4.182122 | C -1.594360 2.570499 0.000000  C -3.074136 2.566417 0.000000  Fe -2.334786 0.924633 0.000000  O -2.335593 3.736412 0.000000 |
| t5 | t6 |
| O -1.984816 2.310195 0.000000  C -3.276613 1.998455 0.731686  C -3.276613 1.998455 -0.731686  Fe -3.276613 3.667822 0.000000 | C -0.227766 1.832972 0.000000  C -0.227766 1.832972 -1.302409  Fe -0.227766 1.832972 -3.085217  O -0.227766 1.832972 -4.630994 |

**Table S21** The Cartesian coordinates (in Angstrom units) of isomers of FeC_2_O in their singlet electronic state obtained at different levels of theory.

| s1; TPSSh/6-311++G(2d,2p) | | | | s1; TPSSh/SDD & 6-311++G(2d,2p) | | | | |
| --- | --- | --- | --- | --- | --- | --- | --- | --- |
| Fe | -1.473876 | -0.238126 | 0.000000 | Fe | 0.191000 | 3.225028 | 0.000000 | |
| C | -2.253407 | 1.065325 | 0.000000 | C | 1.677888 | 2.920661 | 0.000000 | |
| C | 0.131794 | 0.493233 | 0.000000 | C | -0.315591 | 1.546610 | 0.000000 | |
| O | 1.103404 | 1.114065 | 0.000000 | O | -0.522379 | 0.412499 | 0.000000 | |
| s1; ωB97XD /6-311++G(2d,2p) | | | | s1; ωB97XD /SDD & 6311++G(2d,2p) | | | | |
| Fe | -1.608554 | 3. 209970 | 0.000000 | Fe | 0.203370 | 3.224016 | 0.000000 | |
| C | -0.140075 | 2.938193 | 0.000000 | C | 1.667372 | 2.923598 | 0.000000 | |
| C | -2.154830 | 1.516488 | 0.000000 | C | -0.318996 | 1.540156 | 0.000000 | |
| O | -2.336820 | 0.390215 | 0.000000 | O | -0.520828 | 0.417028 | 0.000000 | |
| s2; ωB97XD /6-311++G(2d,2p) | | | | s3; TPSSh/6-311++G(2d,2p) | | | | |
| Fe | -0.811418 | -0.186149 | -0.001001 | Fe | 0.235576 | 0.132164 | 0.650015 | |
| C | 1.450856 | 0.158881 | 0.003608 | C | -0.100272 | -0.052410 | -2.343517 | |
| C | 0.416626 | 0.991421 | 0.005000 | C | 0.044015 | 0.025235 | -1.061851 | |
| O | 2.484517 | -0.391739 | 0.002423 | O | -0.231319 | -0.124578 | -3.515467 | |
| s3; TPSSh/SDD & 6-311++G(2d,2p) | | | | s3; ωB97XD /6-311++G(2d,2p) | | | | |
| Fe | -2.642143 | 0.778520 | 0.000092 | Fe | -3.564551 | 0.400183 | 0.000372 | |
| C | 0.352441 | 0.780226 | -0.001084 | C | -0.549519 | 0.401384 | 0.000536 | |
| C | -0.956423 | 0.780304 | -0.000269 | C | -1.840060 | 0.404728 | 0.000375 | |
| O | 1.519396 | 0.777799 | 0.000452 | O | 0.620962 | 0.399316 | 0.000275 | |
| s3; ωB97XD /SDD & 6311++G(2d,2p) | | | | s4; TPSSh/6-311++G(2d,2p) | | | | |
| Fe | -3.476562 | 0.443582 | 0.000451 | Fe | -1.267258 | -0.084764 | 0.000000 | |
| C | -0.490073 | 0.445013 | -0.000618 | C | -1.074011 | 1.686454 | 0.000000 | |
| C | -1.786149 | 0.448050 | -0.001098 | C | 0.136603 | 1.067640 | 0.000000 | |
| O | 0.671907 | 0.443681 | 0.000456 | O | -2.316026 | 1.541844 | 0.000000 | |
| s4; TPSSh/SDD & 6-311++G(2d,2p) | | | | s4; ωB97XD /6-311++G(2d,2p) | | | | |
| Fe | 0.357316 | -1.222574 | 0.000000 | Fe | -2.136221 | 3.432183 | -0.061745 | |
| C | 0.479613 | 0.555393 | 0.000000 | C | -2.011568 | 1.633080 | -0.061745 | |
| C | 1.714983 | 0.005640 | 0.000000 | C | -0.792782 | 2.135331 | -0.061745 | |
| O | -0.757626 | 0.387140 | 0.000000 | O | -3.240509 | 1.777122 | -0.061745 | |
| s4; ωB97XD /SDD & 6311++G(2d,2p) | | | | s5; TPSSh/6-311++G(2d,2p) | | | | |
| Fe | 0.350152 | -1.268843 | 0.000000 | Fe | -0.000688 | 0.445856 | -0.323158 | |
| C | 0.487361 | 0.538257 | 0.000000 | C | -0.822220 | -1.108010 | 0.540152 | |
| C | 1.702382 | 0.042598 | 0.000000 | C | 1.111568 | 0.912553 | 0.537813 | |
| O | -0.745610 | 0.413588 | 0.000000 | O | -0.257399 | -1.548724 | -0.394807 | |
| s5; TPSSh/SDD & 6-311++G(2d,2p) | | | | s5; ωB97XD /6-311++G(2d,2p) | | | | |
| Fe | -0.024659 | 0.439147 | -0.338036 | Fe | -0.000688 | 0.445856 | -0.323158 | |
| C | -0.817286 | -1.093961 | 0.551588 | C | -0.822220 | -1.108010 | 0.540152 | |
| C | 1.107727 | 0.889967 | 0.543094 | C | 1.111568 | 0.912553 | 0.537813 | |
| O | -0.234521 | -1.533478 | -0.396647 | O | -0.257399 | -1.548724 | -0.394807 | |
| s5; ωB97XD /SDD & 6311++G(2d,2p) | | | | s6; TPSSh/6-311++G(2d,2p) | | | | |
| Fe | -0.005714 | 0.452014 | -0.323304 | Fe | 0.504883 | 1.650822 | -0.349868 | |
| C | -0.821295 | -1.107469 | 0.539432 | C | -0.301688 | 0.627533 | 0.401869 | |
| C | 1.110035 | 0.901818 | 0.537296 | C | 3.298372 | 1.491865 | 0.749909 | |
| O | -0.251765 | -1.544688 | -0.393425 | O | 2.273820 | 1.651730 | 0.244933 | |
| s6; TPSSh/SDD & 6-311++G(2d,2p) | | | | s6; ωB97XD /6-311++G(2d,2p) | | | | |
| Fe | -2.312389 | -2.518775 | 0.000002 | Fe | -2.045969 | -0.241019 | 0.000001 | |
| C | -2.999192 | -1.182820 | -0.000001 | C | -2.743230 | 1.064112 | -0.000001 | |
| C | 0.645581 | -1.939819 | -0.000008 | C | 0.975812 | 0.314385 | -0.000008 | |
| O | -0.461122 | -2.256931 | 0.000002 | O | -0.104847 | -0.028169 | 0.000003 | |
| s6; ωB97XD /SDD & 6-311++G(2d,2p) | | | | s7; TPSSh/6-311++G(2d,2p) | | | | |
| Fe | -2.352738 | -2.501470 | 0.000001 | Fe | -1.036950 | 1.490942 | 0.000000 | |
| C | -3.038850 | -1.191111 | -0.000001 | C | -3.194467 | 0.731691 | 0.000000 | |
| C | 0.677831 | -1.950603 | -0.000007 | C | 0.410368 | 1.105480 | 0.000000 | |
| O | -0.413367 | -2.255161 | 0.000002 | O | -2.257126 | 0.018909 | 0.000000 | |
| s7; TPSSh/SDD & 6-311++G(2d,2p) | | | | s7; ωB97XD /6-311++G(2d,2p) | | | | |
| Fe | -1.021754 | -0.907440 | 0.000000 | Fe | -1.710116 | 2.279015 | 0.000000 | |
| C | -2.286896 | -2.830336 | 0.000000 | C | -2.991302 | 0.304919 | 0.000000 | |
| C | 0.369105 | -0.364282 | 0.000000 | C | -0.328500 | 2.794348 | 0.000000 | |
| O | -1.111334 | -2.825538 | 0.000000 | O | -1.837251 | 0.304304 | 0.000000 | |
| s7; ωB97XD /SDD & 6-311++G(2d,2p) | | | | s8; TPSSh/6-311++G(2d,2p) | | | | |
| Fe | -1.008491 | -0.861382 | 0.000000 | Fe | -4.688775 | 1.290069 | 0.000000 | |
| C | -2.285101 | -2.858788 | 0.000000 | C | -6.203281 | 1.165454 | 0.000000 | |
| C | 0.375556 | -0.361876 | 0.000000 | C | -2.557173 | 1.465459 | 0.000000 | |
| O | -1.132843 | -2.845550 | 0.000000 | O | -1.431074 | 1.558115 | 0.000000 | |
| s8; TPSSh/SDD & 6-311++G(2d,2p) | | | | s8; ωB97XD /6-311++G(2d,2p) | | | | |
| Fe | -3.577586 | -0.689655 | 3.273177 | Fe | 0.000000 | 0.000000 | 0.681626 | |
| C | -3.577586 | -0.689655 | 1.128286 | C | 0.000000 | 0.000000 | 2.166779 | |
| C | -3.577586 | -0.689655 | 4.787279 | C | 0.000000 | 0.000000 | -1.555220 | |
| O | -3.577586 | -0.689655 | -0.001793 | O | 0.000000 | 0.000000 | -2.673952 | |
| s8; ωB97XD/SDD & 6-311++G(2d,2p) | | | | s9; TPSSh/6-311++G(2d,2p) | | | | |
| Fe | -0.215116 | 0.797468 | 3.360794 | Fe | 2.892775 | 1.320936 | 0.593923 | |
| C | -0.215116 | 0.797468 | 4.842037 | C | 1.368637 | 0.332795 | 0.426199 | |
| C | -0.215116 | 0.797468 | 1.126888 | C | 1.498783 | 1.163170 | -0.572186 | |
| O | -0.215116 | 0.797468 | 0.007853 | O | 4.402687 | 0.992550 | 0.514247 | |
| s9; TPSSh/SDD & 6-311++G(2d,2p) | | | | s9; ωB97XD /6-311++G(2d,2p) | | | | |
| Fe | 0.183327 | 0.001372 | -0.249456 | Fe | 0.683341 | -0.399899 | -0.439539 | |
| C | -1.445494 | 0.650599 | 0.247856 | C | -0.982167 | 0.238798 | -0.009881 | |
| C | -1.445155 | -0.654855 | 0.238918 | C | -0.982089 | -1.044591 | -0.018695 | |
| O | 1.569938 | -0.003031 | 0.426712 | O | 2.086244 | -0.403568 | 0.150877 | |
| s9; ωB97XD/SDD & 6-311++G(2d,2p) | | | | s10; TPSSh/6-311++G(2d,2p) | | | | |
| Fe | 0.196367 | 0.001073 | -0.209180 | Fe | -0.039241 | 0.556640 | 0.000000 | |
| C | -1.458935 | 0.640323 | 0.239221 | C | 0.639042 | -1.168095 | 0.000000 | |
| C | -1.458796 | -0.644498 | 0.230417 | C | -0.635639 | -1.198093 | 0.000000 | |
| O | 1.583980 | -0.002814 | 0.403572 | O | -0.075412 | 2.093591 | 0.000000 | |
| s10; TPSSh/SDD & 6-311++G(2d,2p) | | | | s10; ωB97XD /6-311++G(2d,2p) | | | | |
| Fe | -0.039253 | 0.557148 | 0.000000 | Fe | -0.271181 | 0.726311 | 0.005963 | |
| C | 0.638925 | -1.166445 | 0.000000 | C | 0.402319 | -1.001131 | 0.005963 | |
| C | -0.635599 | -1.196440 | 0.000000 | C | -0.862672 | -1.030902 | 0.005963 | |
| O | -0.075323 | 2.089780 | 0.000000 | O | -0.306792 | 2.239473 | 0.005963 | |
| s10; ωB97XD/SDD & 6- 311++G(2d,2p) | | | | s11; TPSSh/6-311++G(2d,2p) | | | | |
| Fe | -0.039035 | 0.547886 | 0.000000 | Fe | -3.585078 | 0.583921 | -1.556089 | |
| C | 0.653764 | -1.196992 | 0.000000 | C | -4.520305 | 1.535257 | -0.314130 | |
| C | -0.648985 | -1.227651 | 0.000000 | C | -3.251902 | 2.156547 | -0.697024 | |
| O | -0.076994 | 2.160800 | 0.000000 | O | -3.229242 | 0.966582 | 0.243590 | |
| s11; TPSSh/SDD & 6-311++G(2d,2p) | | | | s11; ωB97XD /6-311++G(2d,2p) | | | | |
| Fe | -2.651094 | 1.489634 | 0.000000 | Fe | -3.253688 | 3.711639 | 0.000000 | |
| C | -2.657640 | -0.193172 | 0.726838 | C | -3.272993 | 1.958987 | 0.699929 | |
| C | -2.657640 | -0.193172 | -0.726838 | C | -3.272993 | 1.958987 | -0.699929 | |
| O | -1.362297 | 0.129402 | 0.000000 | O | -1.991818 | 2.342651 | 0.000000 | |
| s11; ωB97XD/SDD & 6- 311++G(2d,2p) | | | | s12; TPSSh/6-311++G(2d,2p) | | | | |
| Fe | -2.622430 | 1.541607 | 0.000000 | Fe | -0.772497 | 1.810985 | 0.000000 | |
| C | -2.660069 | -0.239168 | -0.694752 | C | -1.754030 | 0.465593 | 0.000000 | |
| C | -2.660069 | -0.239168 | 0.694752 | C | 0.039087 | 0.354687 | 0.000000 | |
| O | -1.372544 | 0.168064 | 0.000000 | O | -0.926310 | -0.665788 | 0.000000 | |
| s12; TPSSh/SDD & 6-311++G(2d,2p) | | | | s12; ωB97XD /6-311++G(2d,2p) | | | | |
| Fe | -2.048409 | 1.494134 | 0.000000 | Fe | -2.396831 | 1.795711 | 0.000000 | |
| C | -1.152049 | 0.092511 | 0.000000 | C | -3.225229 | 0.335538 | 0.000000 | |
| C | -2.946521 | 0.091889 | 0.000000 | C | -1.568144 | 0.335458 | 0.000000 | |
| O | -2.046715 | -0.985608 | 0.000000 | O | -2.397440 | -0.774871 | 0.000000 | |
| s12; ωB97XD/SDD & 6- 311++G(2d,2p) | | | | s13; TPSSh/6-311++G(2d,2p) | | | | |
| Fe | -2.048840 | 1.539861 | 0.000000 | Fe | -1.032548 | 0.518799 | 0.000000 | |
| C | -1.214182 | 0.085217 | 0.000000 | C | -1.450553 | -1.143050 | 0.000000 | |
| C | -2.883334 | 0.084539 | 0.000000 | C | -2.128391 | -2.421595 | 0.000000 | |
| O | -2.047337 | -1.016691 | 0.000000 | O | -0.786549 | -2.439943 | 0.000000 | |
| s13; TPSSh/SDD & 6-311++G(2d,2p) | | | | s13; ωB97XD /6-311++G(2d,2p) | | | | |
| Fe | -0.919942 | -1.535754 | 0.000000 | Fe | -1.604315 | -0.496910 | 0.000000 | |
| C | -1.246323 | 0.138092 | 0.000000 | C | -1.265130 | 1.182742 | 0.000000 | |
| C | -1.201994 | 1.583935 | 0.000000 | C | -1.365012 | 2.633603 | 0.000000 | |
| O | -2.413930 | 1.008094 | 0.000000 | O | -0.164546 | 2.087747 | 0.000000 | |
| s13; ωB97XD/SDD & 6- 311++G(2d,2p) | | | | s14; TPSSh/6-311++G(2d,2p) | | | | |
| Fe | -0.662214 | -0.762627 | 0.000000 | Fe | 0.597197 | 0.020857 | 0.000000 | |
| C | -0.376018 | 0.915438 | 0.000000 | C | -2.469337 | 0.359878 | 0.000000 | |
| C | -0.499553 | 2.363948 | 0.000000 | C | -1.174814 | 0.216762 | 0.000000 | |
| O | 0.708428 | 1.832291 | 0.000000 | O | 2.133614 | -0.149001 | 0.000000 | |
| s14; TPSSh/SDD & 6-311++G(2d,2p) | | | | s14; ωB97XD /6-311++G(2d,2p) | | | | |
| Fe | -4.727011 | 0.603448 | -3.084415 | Fe | -0.227766 | 1.832972 | -3.082237 | |
| C | -4.727011 | 0.603448 | -0.002077 | C | -0.227766 | 1.832972 | -0.019414 | |
| C | -4.727011 | 0.603448 | -1.304155 | C | -0.227766 | 1.832972 | -1.313675 | |
| O | -4.727011 | 0.603448 | -4.627973 | O | -0.227766 | 1.832972 | -4.603294 | |
| s14; ωB97XD/SDD & 6- 311++G(2d,2p) | | | | s15; TPSSh/6-311++G(2d,2p) | | | | |
| Fe | -4.727011 | 0.603448 | -0.022422 | Fe | -0.002488 | -1.579633 | 0.000000 | |
| C | -4.727011 | 0.603448 | -1.316578 | C | -1.301840 | -0.508326 | 0.000000 | |
| C | -4.727011 | 0.603448 | -3.081164 | C | 1.165337 | -0.366290 | 0.000000 | |
| O | -4.727011 | 0.603448 | -4.598455 | O | -0.111976 | 0.322189 | 0.000000 | |
| s15; TPSSh/SDD & 6-311++G(2d,2p) | | | | s15; ωB97XD /6-311++G(2d,2p) | | | | |
| Fe | -1.300623 | 0.825545 | 1.901922 | Fe | 0.000000 | 0.000000 | 0.628049 | |
| C | -1.300623 | -0.412101 | 0.761049 | C | 0.000000 | 1.219901 | -0.522117 | |
| C | -1.300623 | 2.063192 | 0.761049 | C | 0.000000 | -1.219901 | -0.522117 | |
| O | -1.300623 | 0.825545 | 0.002461 | O | 0.000000 | 0.000000 | -1.258058 | |
| s15; ωB97XD/SDD & 6- 311++G(2d,2p) | | | | s16; TPSSh/6-311++G(2d,2p) | | | | |
| Fe | -1.300623 | 0.825545 | 1.899060 | Fe | -0.628366 | 0.960503 | -4.182122 | |
| C | -1.300623 | 2.046697 | 0.754598 | C | -0.628366 | 0.960503 | -1.277167 | |
| C | -1.300623 | -0.395607 | 0.754598 | C | -0.628366 | 0.960503 | 0.000000 | |
| O | -1.300623 | 0.825545 | 0.018226 | O | -0.628366 | 0.960503 | -2.531972 | |
| s16; TPSSh/SDD & 6-311++G(2d,2p) | | | | s16; ωB97XD /6-311++G(2d,2p) | | | | |
| Fe | -5.014368 | -0.344828 | 4.176831 | Fe | -1.594360 | 2.917570 | 4.167293 |  |
| C | -5.014368 | -0.344828 | 1.278989 | C | -1.594360 | 2.917570 | 1.272867 |  |
| C | -5.014368 | -0.344828 | 0.001949 | C | -1.594360 | 2.917570 | 0.015134 |  |
| O | -5.014368 | -0.344828 | 2.533492 | O | -1.594360 | 2.917570 | 2.535968 |  |
| s16; ωB97XD/SDD & 6- 311++G(2d,2p) | | | | s17; TPSSh/6-311++G(2d,2p) | | | |  |
| Fe | -0.900217 | 0.748373 | 4.159234 | Fe | -0.950188 | 0.189811 | 0.003998 |  |
| C | -0.900217 | 0.748373 | 1.272484 | C | -1.911660 | -1.285898 | 0.000745 |  |
| C | -0.900217 | 0.748373 | 0.015361 | C | -0.697194 | -1.729101 | -0.002395 |  |
| O | -0.900217 | 0.748373 | 2.536387 | O | -1.420688 | 1.652055 | -0.002349 |  |
| s17; TPSSh/SDD & 6-311++G(2d,2p) | | | | s17; ωB97XD/SDD & 6- 311++G(2d,2p) | | | |  |
| Fe | 0.173042 | -0.815388 | -0.000371 | Fe | 0.169757 | -0.832641 | -0.000353 |  |
| C | -1.599044 | -1.820377 | -0.000952 | C | 1.621767 | -1.824693 | -0.000817 |  |
| C | 2.129317 | -0.641713 | -0.006275 | C | 2.099607 | -0.640209 | -0.006362 |  |
| O | -1.291050 | -1.264674 | -0.005301 | O | -1.280777 | -1.244610 | -0.005367 |  |
| s18; TPSSh/6-311++G(2d,2p) | | | | s18; TPSSh/SDD & 6-311++G(2d,2p) | | | |  |
| Fe | -4.176780 | 0.650470 | -0.171805 | Fe | -1.665838 | -0.200782 | 0.037015 |  |
| C | -2.603281 | 0.979672 | 0.001650 | C | -3.257846 | -0.200823 | 0.045528 |  |
| C | -0.126657 | 1.497822 | 0.274473 | C | -5.845356 | -0.200890 | 0.059365 |  |
| O | -1.284415 | 1.255601 | 0.147072 | O | -4.673910 | -0.200860 | 0.053101 |  |
| s19; TPSSh/6-311++G(2d,2p) | | | | s20; TPSSh/6-311++G(2d,2p) | | | |  |
| Fe | 0.200912 | 1.014715 | -0.416811 | Fe | -2.191305 | 1.232444 | 0.000000 |  |
| C | 1.836539 | 1.426976 | 0.358988 | C | -0.532349 | 1.943057 | 0.000000 |  |
| C | -1.079481 | 0.843967 | 0.371647 | C | -0.563126 | 0.463596 | 0.000000 |  |
| O | 2.928896 | 1.674482 | 0.639501 | O | 0.620002 | 1.180912 | 0.000000 |  |
